# Computing a Link Diagram From Its Exterior

**DOI:** 10.1007/s00454-023-00533-w

**Published:** 2023-08-02

**Authors:** Nathan M. Dunfield, Malik Obeidin, Cameron Gates Rudd

**Affiliations:** 1 Deptment of Mathematics, University of Illinois Urbana-Champaign, 1409 W. Green Street, Urbana, IL 61801 USA; 2 Google, Inc., Atlanta, GA USA; 3 Max-Planck-Institut für Mathematik, Bonn, Germany

**Keywords:** Computational topology, Low-dimensional topology, Knot, Knot exterior, Knot diagram, Link, Link exterior, Link diagram, 57K10, 57K30

## Abstract

A knot is a circle piecewise-linearly embedded into the 3-sphere. The topology of a knot is intimately related to that of its exterior, which is the complement of an open regular neighborhood of the knot. Knots are typically encoded by planar diagrams, whereas their exteriors, which are compact 3-manifolds with torus boundary, are encoded by triangulations. Here, we give the first practical algorithm for finding a diagram of a knot given a triangulation of its exterior. Our method applies to links as well as knots, and allows us to recover links with hundreds of crossings. We use it to find the first diagrams known for 23 principal congruence arithmetic link exteriors; the largest has over 2500 crossings. Other applications include finding pairs of knots with the same 0-surgery, which relates to questions about slice knots and the smooth 4D Poincaré conjecture.

## Introduction

**Fig. 1:**
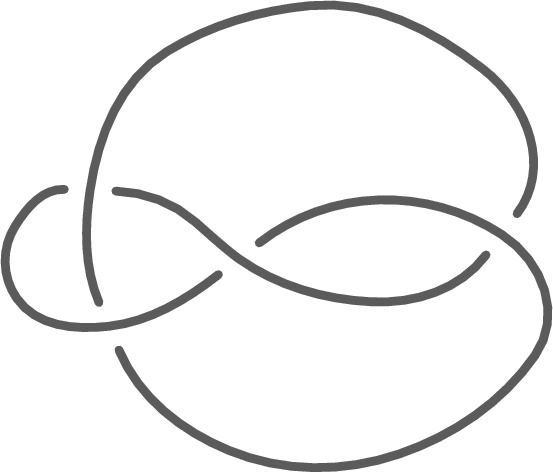
A planar diagram for a knot can be viewed as a 4-valent graph (the “shadow” of the above figure) with a planar embedding where every vertex represents a *crossing*, a place where one part of the knot crosses in front of the other in 3D

A knot is a piecewise-linear (PL) embedding of a circle $S^1$ into the 3-sphere $S^3$. The study of knots goes back to the 19th century, and today is a central focus of low-dimensional topology, with applications to chemistry [25], biology [26], engineering [55], and theoretical computer science [47]. Two knots are topologically equivalent when they are isotopic, that is, when one can be continuously deformed to the other without passing through itself. Computationally, knots are typically encoded as planar diagrams (Fig. 1); there are more than 350 million distinct knots with diagrams of at most 19 crossings as enumerated by [12].

The topology of knots is intimately related to that of their exteriors, where the *exterior* of a knot *K* is the compact 3-manifold with torus boundary $E(K):=S^3\setminus N(K)$, where *N*(*K*) is an open tubular neighborhood of *K*. Indeed, the orientation-preserving homeomorphism type of the exterior $E(K)$ determines the knot *K* [28]. Many algorithms for knots work via their exteriors, starting with Haken’s foundational method for deciding when a knot is equivalent to the unknot [30]. Consequently, the problem of going from a diagram *D* of *K* to a triangulation of $E(K)$ is well studied [31, Sect. 7]; for ideal triangulations (see Sect. 2.1 below), one needs only four tetrahedra per crossing of *D* [71, Sect. 3]. Here, we study the inverse problem:

**FIND DIAGRAM** Input a triangulation ${\mathcal T}$ of a knot exterior $E(K)$, output a diagram of *K*.

**Fig. 2:**
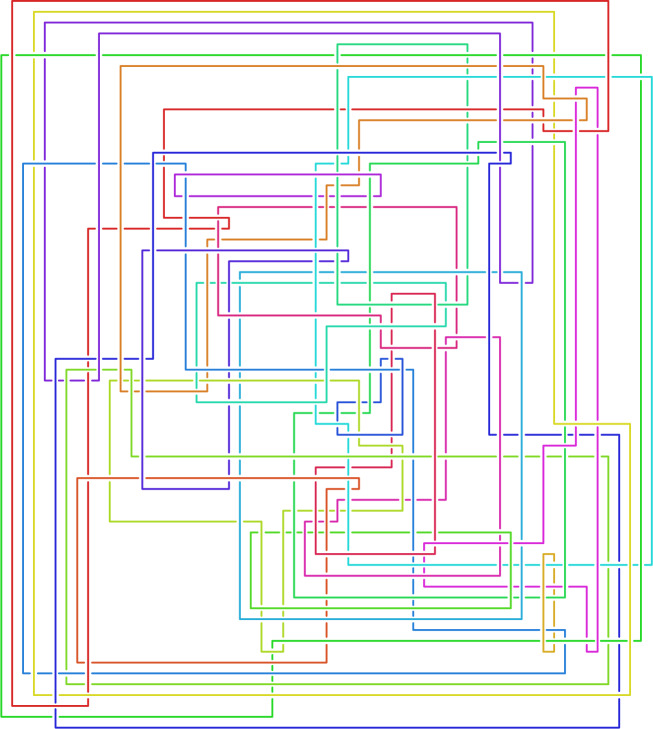
The first known diagram of a link whose exterior is $\mathring{M}={{\mathbb {H}}^3}/\Gamma (I)$ where $\Gamma (I)$ is the principal congruence subgroup of $\textrm{PSL}_{2} {\mathbb Z}\bigl [\bigl (1+i\sqrt{15}\bigr )/2\bigr ]$ of level $I=\bigl \langle 6,\bigl (-3+i\sqrt{15}i\bigr )/2\bigr \rangle$ from [6]; it has 24 components and 294 crossings. The input ideal triangulation $\mathring{{\mathcal T}}$ for $\mathring{M}$ had 249 tetrahedra. Since the hyperbolic volume of $\mathring{M}\approx 225.98$, any diagram must have at least 66 crossings by [3, Thm. 5.1]

**Fig. 3:**
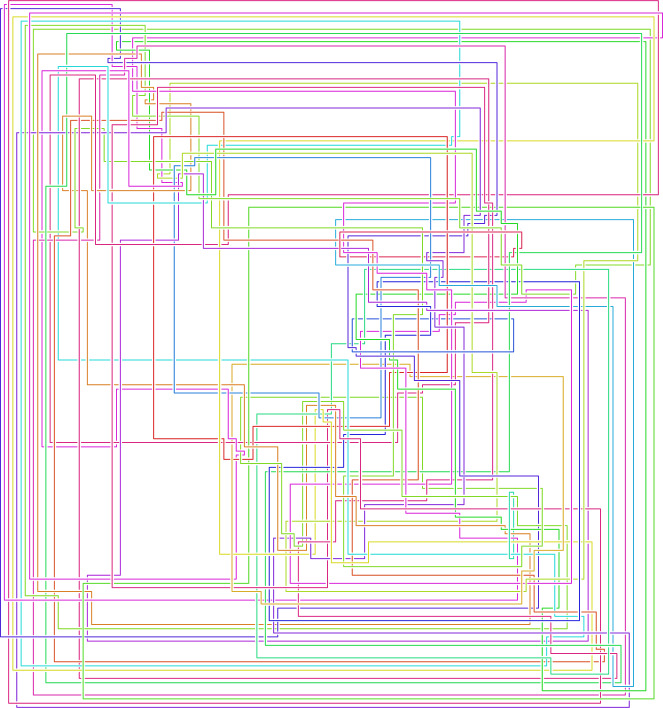
The first known diagram of a link whose exterior is $\mathring{M}={\mathbb {H}}^3/\Gamma (I)$ where $\Gamma (I)$ is the principal congruence subgroup of $\textrm{PSL}_{2} {\mathbb Z}\bigl [\bigl (1+i\sqrt{15}\bigr )/2\bigr ]$ of level $I={\bigl \langle 5,\bigl (5+i\sqrt{15}\bigr )/2 \bigl \rangle }$ from [6]; it has 24 components and 1092 crossings. The input ideal triangulation $\mathring{{\mathcal T}}$ for $\mathring{M}$ had 211 tetrahedra. Since the hyperbolic volume of $\mathring{M}\approx 188.32$, any diagram must have at least 56 crossings [3, Thm. 5.1]

If the input triangulation ${\mathcal T}$ is guaranteed to be that of a knot exterior (in fact, this is decidable by [40, Algorithm S]), then a useless algorithm to find *D* is just this: start generating all knot diagrams, triangulate each exterior, and then do Pachner moves (see Sect. 2.4) on these triangulations. Since any two triangulations of a compact 3-manifold are connected by a sequence of such moves, one eventually stumbles across ${\mathcal T}$, thus finding a diagram for the underlying knot. We do not explore the computational complexity of Find Diagram  beyond showing it is at least exponential space in Theorem 9.1, but rather give the first algorithm that is highly effective in practice. We work more generally with links, where a *link* is a disjoint union of knots. While a link exterior does not uniquely determine a link [1, Fig. 9.28], this indeterminacy is removed by specifying meridional curves for the link, see Sect. 2.3; hence we require such curves as part of the input in Sect. 1.2. Figures 2  and 3  show diagrams that were found by our method; these are the first known diagrams of these particular link exteriors, see Sect. 11.1.

### Prior Work

In general, an efficient algorithmic solution to the homeomorphism problem has not been implemented, and would be quite complicated; see [42]. However, when the interior of $E(K)$ has a complete hyperbolic structure, in short is *hyperbolic*, the homeomorphism problem can be quickly solved in practice using hyperbolic geometry, even for triangulations with 1000 tetrahedra [69]. This case is in practice generic for prime knots; for example, $99.999\,\%$ of the knots in [12] are hyperbolic. This allows a table lookup method for Find Diagram when *K* is small enough; one uses hyperbolic and homological invariants to form a hash of $E(K)$, queries a database of knots to get a handful of possible $K_i$, and then checks if any $E(K_i)$ is homeomorphic to $E(K)$. This technique is used by the identify method of [18], but is hopeless for something like Fig. 2, as the number of links of that size exceeds the number of atoms in the visible universe [65].

A related approach was used in [5, 17] to find knot diagrams for all 1267 knots where $E(K)$ is hyperbolic and can be triangulated with at most nine ideal tetrahedra [13, 19]. While knots with few crossings have simple exteriors, the converse is not the case, and the simplest known diagrams for about $25\,\%$ of these knots have 100–300 crossings. However, these knots either fall into very special families which can be tabulated to a large number of crossings, or one can drill out additional curves to get a link exterior that appears in an existing table and has special properties allowing the recovery of a diagram of the knot itself.

The exteriors of the special class of alternating knots have nice topological characterizations given in [29] and [34]. Using these characterizations, Howie [34] and independently Juhász and Lackenby in an appendix to [29] describe normal surface theory algorithms for determining whether a 3-manifold $E(K)$ is the exterior of an alternating knot. The certificate that $E(K)$ is the exterior of an alternating knot can then be used to produce an alternating knot diagram of *K*; see [34, p. 2370].

There are other ad hoc methods in the literature, see e.g. [7] and references therein, but this paper is the first to give a generically applicable method for Find Diagram.

### Outline of the Algorithm

As Figs. 2 and 3 show, our method can solve Find Diagram in cases where any diagram for the link has 66 and 55 or more crossings respectively. It also easily handles any example covered by one of the techniques discussed in Sect. 1.1, and more applications are given in Sects. 10 and 11. Experimental mean running time was $O(1.07^n)$, where *n* is the number of tetrahedra in the input ideal triangulation, see Fig. 24. With the definitions of Sect. 2, the input for our algorithm is:

*Input*: (a) An ideal triangulation $\mathring{{\mathcal T}}$ of a compact $3$-manifold $\mathring{M}$ with toroidal boundary, with an essential simple closed curve $\alpha _i$ for each boundary component of $\mathring{M}$.
  (b) A sequence $(P_i)$ of Pachner moves transforming the layered filling triangulation ${\mathcal T}$ of the manifold $M=\mathring{M}(\alpha _1,\dots ,\alpha _k)$ into a specific $2$-tetrahedra *base triangulation* ${\mathcal T}_0$ of $S^3$.

One might object that (b) is effectively cheating, since no polynomial-time algorithm for finding $(P_i)$ is known, or indeed for deciding if *M* is $S^3$. Using the estimates in [48], one can perform a naive search to find some $(P_i)$, but the complexity of this is super-exponential. However, recognizing $S^3$ by finding such moves is easy in practice, see Sect. 4, with the length of $(P_i)$ linear in the size of ${\mathcal T}$ as per Fig. 26. The output of the algorithm is a knot diagram *D*, encoded as a planar graph with over/under crossing data for the vertices.

The main data structure is a triangulation ${\mathcal T}$ of $S^3$ with a PL link *L* that is disjoint from the 1-skeleton. The link *L* is encoded as a sequence of line segments, each contained in a single tetrahedron of ${\mathcal T}$, with endpoints recorded in barycentric coordinates. An initial pair $({\mathcal T}, L)$ in (b) is constructed from input (a) as described in Sect. 3. The algorithm proceeds by performing the Pachner moves $P_i$ from (b), keeping track of the PL arcs encoding the link *L* throughout using the techniques of Sect. 5. The result is the base triangulation enriched with PL arcs representing the link *L*. As detailed in Sect. 7, this triangulation of $S^3$ can be cut open along faces and embedded in ${\mathbb R}^3$, giving an embedding of the cut-open link into ${\mathbb R}^3$ as a collection of PL arcs with endpoints on the boundary of these tetrahedra. As in Figs. 20 and 21, these PL arcs are then tied up using the face identifications to obtain a collection of closed PL curves that represent *L*. An initial link diagram *D* is obtained by projecting this PL link onto a plane and recording crossing information. We then apply generic simplification methods to *D* and output the result.

This outline turns out to be deceptively simple. Some key difficulties are:

- Understanding what $2\,{\rightarrow }\,3$ and $3\,{\rightarrow }\,2$ Pachner moves do to the link *L* is fairly straightforward as these correspond to changing the triangulation of a convex polyhedron in ${\mathbb R}^3$. However, while these two moves theoretically suffice for (b), in practice one wants to use $2\,{\rightarrow }\,0$ moves as well, see Sect. 4, and these are much harder to deal with, as Fig. 8  shows. We thus expand each $2\,{\rightarrow }\,0$ move into a (sometimes quite lengthy) sequence of $2\,{\rightarrow }\,3$ and $3\,{\rightarrow }\,2$ moves as discussed in Sect. 6. We give a simplified expansion for the trickiest part, the endpoint-through-endpoint move, using six of the basic $2\,{\rightarrow }\,3$ and $3\,{\rightarrow }\,2$ moves instead of 14.
- The complexity of the link grows very rapidly as we do Pachner moves, resulting in enormously complicated initial diagrams. We greatly reduce this by elementary local simplifications to the link after each Pachner move, see Sect. 5.5.
- Prior work on simplifying link diagrams was focused on those with 30 or fewer crossings, where random application of Reidemeister moves (plus flypes) are extremely effective. Here, we need to simplify diagrams with 10, 000 or even 100, 000 crossings down to something with less than 100, and such methods proved ineffective for this. Instead, we used the more global *strand pickup* method of Sect. 8.

## Background

### Triangulations

Let *M* be a compact orientable $3$-manifold, possibly with boundary. A *triangulation* of *M* is a cell complex ${\mathcal T}$ made from finitely many tetrahedra by gluing some of their $2$-dimensional faces in pairs via orientation-reversing affine maps so that the resulting space is homeomorphic to *M*. These triangulations are not necessarily simplicial complexes, but rather what are sometimes called semi-simplicial, pseudo-simplicial, or singular triangulations. Of particular importance are those with a single vertex, the *1-vertex triangulations*. For any triangulation, we use ${\mathcal T}^i$ to denote the *i*-skeleton of ${\mathcal T}$, that is, the union of cells of dimension at most *i*.

When *M* has nonempty boundary, an *ideal triangulation* of *M* is a cell complex ${\mathcal T}$ made out of finitely many tetrahedra by gluing *all* of their $2$-dimensional faces in pairs as above so that $M\setminus \partial M$ is homeomorphic to ${\mathcal T}\setminus {\mathcal T}^0$. Put another way, the manifold *M* is what you get by gluing together *truncated* tetrahedra in the corresponding pattern. See [67] for background on ideal triangulations, which we use only for $3$-manifolds whose boundary is a union of tori. We always include the modifier “ideal”, so throughout “triangulation” means a non-ideal, also called “finite”, triangulation.

### Triangulations with PL Curves

Consider a tetrahedron $\Delta$ in ${\mathbb R}^n$ as the convex hull of its vertices $v_0$, $v_1$, $v_2$, and $v_3$. We encode points in $\Delta$ using *barycentric coordinates*, that is, write $p\in \Delta$ as the unique convex combination $\sum _ix_iv_i$ and then represent *p* by the vector $(x_0,x_1,x_2,x_3)$, where of necessity $\sum _ix_i=1$. For a 3-manifold triangulation ${\mathcal T}$, we view each tetrahedron $\tau$ as having a fixed identification with the tetrahedron in ${\mathbb R}^4$ whose vertices are the standard basis vectors; we use this to encode points in $\tau$ by barycentric coordinates.

An oriented PL curve in ${\mathcal T}$ will be described by a sequence of such barycentric coordinates as follows. A *barycentric arc* *a* is an ordered pair of points (*u*, *v*) in a tetrahedron $\tau$, representing the straight segment joining them. We write $a.\mathtt{{start}}=u$ and $a.\mathtt{{end}}=v$. A *barycentric curve* *C* is a sequence of barycentric arcs $a_i$ such that $a_i.\mathtt{{end}}$ and $a_{i+1}.\texttt{start}$ correspond to the same point in *M* under the face identifications of ${\mathcal T}$. For a barycentric curve, we define $a_i.\mathtt{{next}}=a_{i+1}$ and $a_{i+1}.\texttt{past}=a_i$; these may not lie in the same tetrahedron. Suppose the barycentric curve *C* consists of *N* barycentric arcs. If $a_0.\mathtt{{start}}$ and $a_N.\texttt{end}$ correspond to the same point in *M*, we have a *barycentric loop*. An embedded barycentric loop is a *barycentric knot*. A *barycentric link* is a finite disjoint union of such knots.

We always require that a barycentric curve *C* is in the following kind of general position with respect to ${\mathcal T}$. First, *C* is disjoint from ${\mathcal T}^1$. Second, any intersection of a constituent barycentric arc *a* with ${\mathcal T}^2$ is an endpoint of *a*. Finally, arcs do not bounce off faces of ${\mathcal T}^2$, so if an arc ends in a face, the next arc must be in the adjacent tetrahedron on the other side of that face. Throughout, we use only points whose barycentric coordinates are in ${\mathbb Q}$.

### Dehn Filling

Suppose $\mathring{M}$ is a compact $3$-manifold whose boundary is a union of tori. A simple closed curve on a surface is *essential* if it does not bound a disk. Given an essential simple closed curve $\alpha _i$ on each boundary component $T_i$, the *Dehn filling* of $\mathring{M}$ along $\alpha =(\alpha _1,\dots ,\alpha _k)$ is the closed 3-manifold $\mathring{M}(\alpha )$ obtained from $\mathring{M}$ by gluing a solid torus $D^2\times S^1$ to each $T_i$ so that $\partial D^2\times \{point \}$ is $\alpha _i$. When $\mathring{M}$ is the exterior of a link *L* in $S^3$ and each $\alpha _i$ is a small meridional loop about the *i*-th component of *L*, then $\mathring{M}(\alpha )$ is just $S^3$. Given an ideal triangulation $\mathring{{\mathcal T}}$ of $\mathring{M}$ and Dehn filling curves $\alpha$, we follow [39, 40, 70] to create a 1-vertex triangulation ${\mathcal T}$ of $\mathring{M}(\alpha )$ that we call the *layered filling triangulation*; see Sect. 3. A key point is that the link *L* consisting of the cores of the *k* added solid tori is a barycentric link in ${\mathcal T}$ made of just *k* barycentric arcs.

### Pachner Moves

**Fig. 4:**
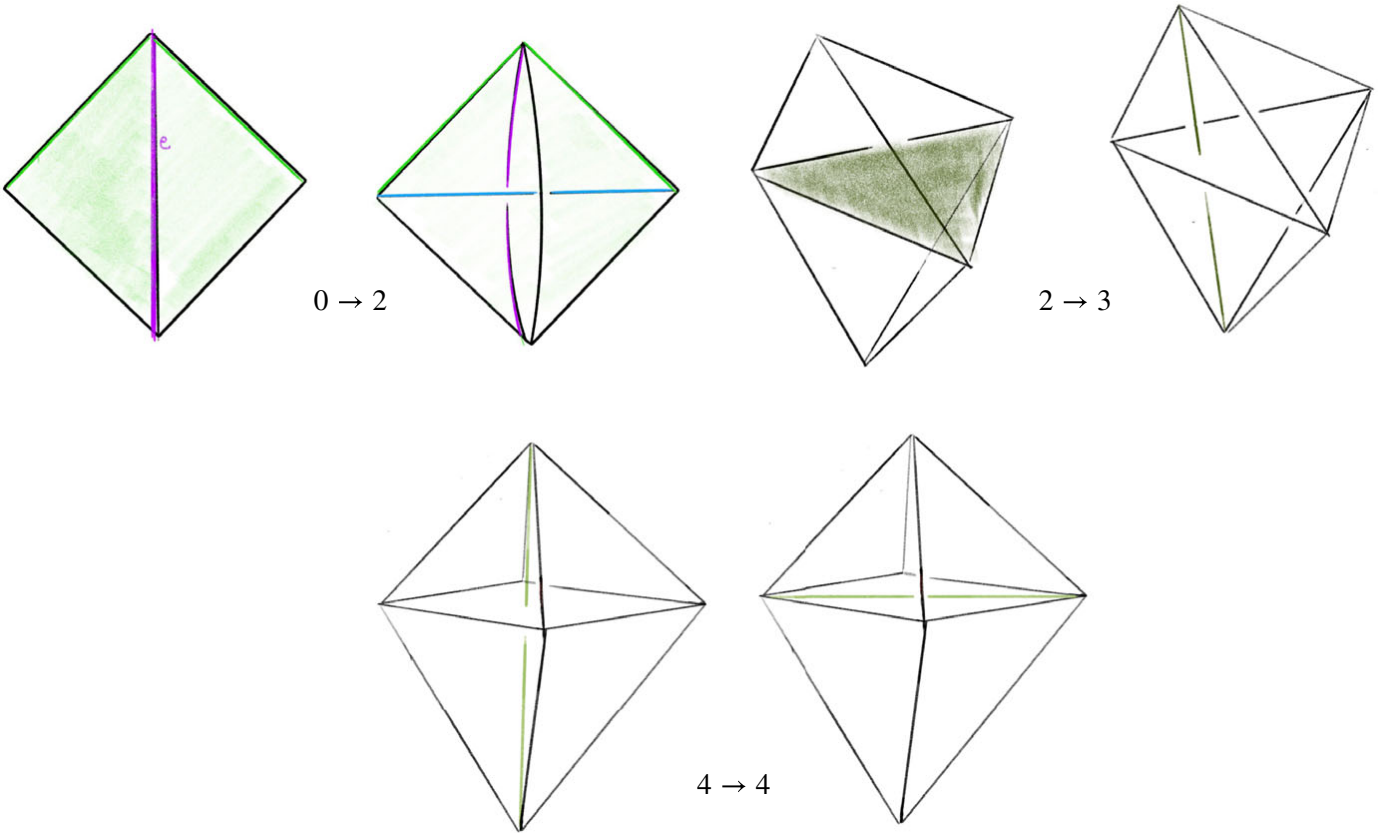
Pachner moves that preserve the number of vertices

A 3-manifold triangulation ${\mathcal T}$ can be modified by local *Pachner moves*, also known as *bistellar flips* to give a new triangulation of the same underlying manifold. Those we use are shown in Fig. 4   and are as follows:

- The $2\,{\rightarrow }\,3$ move and its inverse $3\,{\rightarrow }\,2$ move. These take a triangulation of a ball, possibly with boundary faces glued together, and retriangulate the interior without changing the boundary triangulation. Specifically, the $2\,{\rightarrow }\,3$ move takes a pair of distinct tetrahedra sharing a face and replaces them with three new tetrahedra around a new central edge. The $3\,{\rightarrow }\,2$ move reverses this, replacing three distinct tetrahedra around a valence-3 edge with two tetrahedra sharing a face.
- The $4\,{\rightarrow }\,4$ move. The $4\,{\rightarrow }\,4$ move takes four tetrahedra around a central edge and replaces them with four new tetrahedra assembled around a new valence-4 edge.
- The $2\,{\rightarrow }\,0$ move and its inverse $0\,{\rightarrow }\,2$ move. The $2\,{\rightarrow }\,0$ move takes a pair of tetrahedra sharing two faces to form a valence-2 edge and collapses them onto their common faces. The $0\,{\rightarrow }\,2$ move reverses this by puffing air into a pair of faces sharing an edge and adding two new tetrahedra. We call the complex created by the $0\,{\rightarrow }\,2$ move a *pillow*. The $0\,{\rightarrow }\,2$ move inflates a pillow and the $2\,{\rightarrow }\,0$ move collapses a pillow.

If ${\mathcal S}$ and ${\mathcal T}$ are two $1$-vertex triangulations of the same closed 3-manifold *M*, then there is a sequence of Pachner moves that transforms ${\mathcal S}$ into ${\mathcal T}$, provided both ${\mathcal S}$ and ${\mathcal T}$ have at least two tetrahedra. To do this, one need only use $2\,{\rightarrow }\,3$ and $3\,{\rightarrow }\,2$ moves by [46, Thm. 1.2.5] (see also [54, 57]). As noted in the introduction, the $2\,{\rightarrow }\,0$ and $0\,{\rightarrow }\,2$ moves are much harder to deal with than the others. We will call the $2\,{\rightarrow }\,3$, $3\,{\rightarrow }\,2$, and $4\,{\rightarrow }\,4$ moves the *simple Pachner moves*, and note that one needs only these moves to connect two triangulations as above. However, as discussed in Remark 4.1, the $2\,{\rightarrow }\,0$ and $0\,{\rightarrow }\,2$ moves are extremely useful in practice. When *M* is $S^3$, any triangulation ${\mathcal T}$ with *n* tetrahedra is related to a standard triangulation by at most $12\cdot 10^6n^22^{2\cdot 10^3n^2}$ Pachner moves [48]. Experimentally, one needs many fewer moves [10]. In our data shown in Fig. 26, the number is *O*(*n*); this is essential for the utility of our algorithm for Find Diagram.

## Building the Initial Triangulation

In this section, we detail the construction of the layered filling triangulation ${\mathcal T}$, mentioned in Sect. 2.3, from part (a) of the input: an ideal triangulation $\mathring{{\mathcal T}}$ and Dehn filling slopes $\alpha$. This procedure is nearly identical to the approach used in the SnapPy kernel [70] for constructing triangulations of Dehn fillings, with a slight tweak at the very last step to end up with a triangulation in the style of [40] containing layered triangulations of the Dehn filling solid tori.

**Fig. 5:**
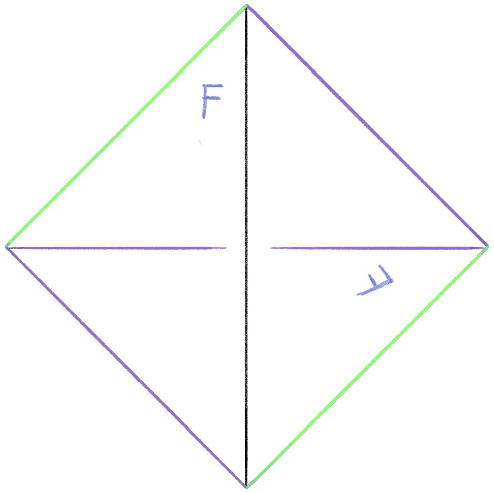
A 1-tetrahedron triangulation of a solid torus. Here, the back two faces are identified with the twist indicated by the letter “F”; the edge colors indicate the equivalence classes in the glued-up triangulation [35, Appendix A]

**Fig. 6:**
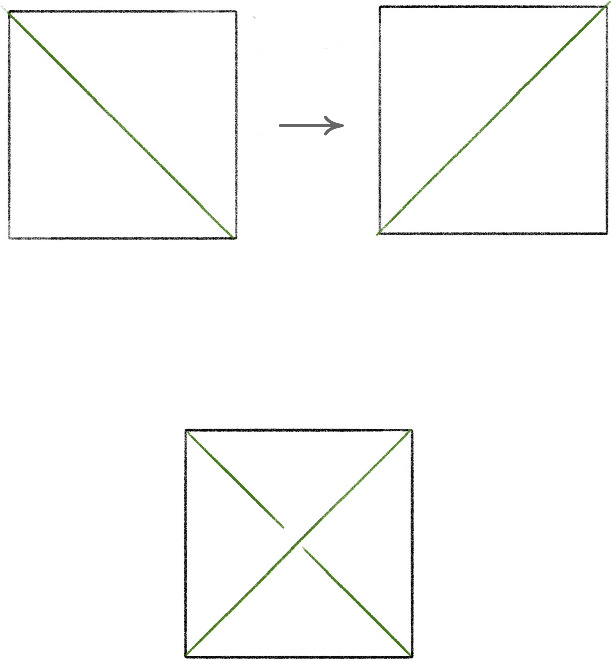
An edge flip (top) and corresponding tetrahedron (bottom)

Given a single tetrahedron, the face identification indicated in Fig. 5   produces a solid torus. Any $1$-tetrahedron triangulation of a solid torus is combinatorially equivalent to this one. The triangulation of the boundary torus induced by the $1$-tetrahedron triangulation of the solid torus is the standard $1$-vertex triangulation of a torus. For any edge *e* of the boundary torus, there is a move modifying the triangulation that, after cutting open the torus so that the edge *e* is the diagonal set inside a square, flips the diagonal. This is commonly called an *edge flip* move. Any such flip move can be realized by attaching a tetrahedron as in Fig. 6 ; this produces a triangulation of a solid torus with an additional tetrahedron and with boundary triangulated according to the flip move. A layered solid torus with *t* layers is a triangulation of a solid torus that is obtained from a $(t-1)$-layer layered solid torus by attaching a new tetrahedron realizing some bistellar flip of the boundary torus. A 0-layer layered solid torus is the $1$-tetrahedron solid torus. This 0-layer solid torus contained in the layered solid torus is called the *core solid torus*. While every layered solid torus has boundary given by the standard one vertex triangulation of the torus, the isotopy class, or *slope*, of the boundary of a meridian disk changes as layers are added.

Let *T* be the standard triangulation of a torus and $\alpha$ a slope on *T*. There is an algorithm for producing a layered solid torus so that filling $\alpha$ bounds a meridian disk, see [40, Thm. 4.1]. One can then attach this layered solid torus to a triangulated manifold $\mathring{M}$ whose torus boundary is triangulated in the standard $1$-vertex way to obtain a triangulation of the Dehn filling $\mathring{M}(\alpha )$. We build the layered filling triangulation from $\mathring{{\mathcal T}}$ and $\alpha$ as follows. For notational simplicity, we assume $\mathring{M}$ has only one boundary component.

### Algorithm 3.1

$$\mathtt{layered\_filling\_triangulation}(\mathring{{\mathcal T}},\alpha )$$

1. Truncate the ideal tetrahedra of $\mathring{{\mathcal T}}$ to obtain a cell complex homeomorphic to the compact manifold with torus boundary $\mathring{M}$.
2. Subdivide this cell complex by placing a vertex at the center of each hexagonal face to divide it into six triangles, and then coning to the middle of every 3-cell. This produces a triangulation of $\mathring{M}$.
3. Simplify the triangulation of the boundary using the procedure in [70], which largely consists of folding two adjacent triangles across their common edge, until the boundary tori are triangulated in the standard way.
4. Add layers to the boundary until the slope $\alpha$ is standard, that is, corresponds to the meridian curve of the 0-layer solid torus.
5. Attach the 0-layer solid torus.
6. Collapse edges joining distinct vertices to obtain a $1$-vertex triangulation of $M(\alpha )$.

Note Algorithm 3.1 is identical to [70] except for step 5, where instead one adds a single tetrahedron with two faces folded together to form a valence-1 edge; with vertices identified, this single tetrahedron is a solid torus with a meridian disk collapsed to a point.

Because we constructed the layered filling triangulation ${\mathcal T}$ from an ideal triangulation of a manifold with toroidal boundary, we know which layered solid tori come from Dehn filling. For each such layered solid torus, its core curve can be represented by the line segment running between the barycenters of the faces in the core solid torus that are glued together. In particular, there are natural barycentric arcs that represent the link *L* consisting of the core curves of all the Dehn fillings. We add these arcs to produce the initial triangulation ${\mathcal T}$ of $M(\alpha )$ with its associated barycentric link.

## Finding Certificates

Part (b) of the input to our algorithm is a certificate that the Dehn filling $M=\mathring{M}(\alpha )$ is $S^3$ in the form of Pachner moves simplifying a triangulation ${\mathcal T}$ of *M* to the base triangulation ${\mathcal T}_0$ of $S^3$. In practice, one starts with an ideal triangulation $\mathring{{\mathcal T}}$ and Dehn filling slopes $\alpha$ where it is unknown if $M(\alpha )$ is $S^3$. We therefore need a way of finding this sequence of Pachner moves when it exists. While deciding if a closed 3-manifold *M* is $S^3$ is in $\textbf{NP}$ by [36, 60] and additionally in ${{\textbf {co-NP}}}$ assuming the Generalized Riemann Hypothesis [72, Thm. 11.2], no sub-exponential time algorithm is known. The current algorithm that is best in practice for $S^3$ recognition is to first heuristically simplify the input triangulation using Pachner moves and then apply the theory of almost normal surfaces; see [11, Algorithm 3.2]. However, triangulations of $S^3$ that are truly hard to simplify using Pachner moves have not been encountered in practice, and it is open whether they exist at all [10]. Thus, when *M* is $S^3$, the initial stage of [11, Algorithm 3.2] nearly always arrives at a 1-tetrahedron triangulation of $S^3$ and no normal surface theory is needed. The usefulness of our algorithm for Find Diagram relies on the fact that a heuristic search using Pachner moves gives a practical recognition algorithm for $S^3$.

### Remark 4.1

The effectiveness of our heuristic search procedure relies on the $2\,{\rightarrow }\,0$ move being atomic. Initially, we tried restricting our heuristic search to just the *simple* Pachner moves (recall these are $2\,{\rightarrow }\,3$, $3\,{\rightarrow }\,2$, and $4\,{\rightarrow }\,4$), but were typically unable to find a sequence that simplified the input triangulation of $S^3$ down to one with just a few tetrahedra. (To square this with [10], note from Fig. 26 that our triangulations are much larger.) As is clear from Sect. 6.1, factoring the $2\,{\rightarrow }\,0$ move as a sequence of $2\,{\rightarrow }\,3$ and $3\,{\rightarrow }\,2$ moves is complicated enough that one cannot expect to stumble upon these sequences when the triangulation is large and the search is restricted to simple Pachner moves.

Our simplification heuristic closely follows that of SnapPy [18], with some modifications that reduce the complexity of the final barycentric link in ${\mathcal T}_0$. These include:

- Simplifying the layered filling triangulation ${\mathcal T}$ of Sect. 2.3 as much as possible without modifying the few tetrahedra containing the initial link.
- Finding sequences of Pachner moves to ${\mathcal T}_0$ for several different layered filling triangulations, and then using the one requiring the fewest moves for the computations in Sects. 5–8.
- Ensuring the tail of the sequence of moves is a geodesic in the Pachner graph of [10].

The details are in Sect. 4.1.

### Basic Triangulation Simplification

In this section, we detail how the initial triangulation ${\mathcal T}$ and Pachner moves transforming it into ${\mathcal T}_0$ are constructed from the input pair $(\mathring{{\mathcal T}}, \alpha )$. Our overall goal is to minimize the number of Pachner moves and, especially, minimize the number that involve any arcs.

The SnapPy kernel [18] provides two routines for trying to simplify a triangulation. The first is simplify, which greedily does various moves that immediately reduce the number of tetrahedra, as well as random $4\,{\rightarrow }\,4$ moves in hopes of setting up such a reduction; it is very similar to [11, Alg. 2.5] which is intelligentSimplify in Regina [15]. The second is randomize, which first does 4*t* random $2\,{\rightarrow }\,3$ moves, where *t* is the number of tetrahedra, and then calls simplify; it is key for escaping local minima in the set of triangulations. In practice, one sometimes needs randomize in order to reduce a layered filling triangulation ${\mathcal T}$ to ${\mathcal T}_0$. Because randomize increases the number of tetrahedra drastically, however temporarily, we work hard to avoid applying it when there are arcs present. We modified simplify and randomize so that one can specify a subcomplex of the triangulation that is to remain unchanged. Our basic strategy is:

1. Construct the layered filling triangulation ${\mathcal T}$ from $(\mathring{{\mathcal T}},\alpha )$.
2. Apply simplify and randomize extensively to ${\mathcal T}$ with the proviso that each tetrahedron that is the core of a filling layered solid torus is not modified. Call the new triangulation ${\mathcal T}'$. It contains a barycentric link $L'$ consisting of the cores of the layered solid tori, which is isotopic to the original *L* in ${\mathcal T}$.
3. If simplify reduces ${\mathcal T}'$ to ${\mathcal T}_0$, record the sequence of Pachner moves and consider $({\mathcal T}',L, (P_i))$ a candidate input for the core algorithm. Otherwise, throw it away.
4. Go back to 1. until we have several candidates for $({\mathcal T}',L,(P_i))$ or we get tired. If no candidate is found, raise an error; otherwise output the one where $(P_i)$ is shortest.

Despite needing randomize to simplify some triangulations of $S^3$ to ${\mathcal T}_0$, so far the above has always succeeded.

Finally, it turns out the last few Pachner moves are the most expensive, since the link is usually quite complicated at that point. Therefore, we built a look-up table of all triangulations of $S^3$ with at most five tetrahedra, along with geodesic Pachner move sequences reducing these triangulations to ${\mathcal T}_0$. If, when searching for a sequence of Pachner moves, we reduce the initial triangulation to one with fewer than five tetrahedra, we can look up whether we indeed have $S^3$, and we then append to the certificate the geodesic Pachner move sequence reducing to ${\mathcal T}_0$. While this only shortens the sequence by a few moves, it gave us a major speedup. For further details, see the file simplify_to_base_tri.py in [20]. One referee points out that we might be able to do even better with a larger look-up table. Currently, it contains 1448 triangulations, and adding six or seven tetrahedra would increase this by 13, 660 and 169, 077 triangulations respectively [10, Sect. 3.1]. However, since the simplification heuristic of Sect. 4 greedily applies $3\,{\rightarrow }\,2$ moves whenever they are available, one only needs store geodesics for those triangulations where either there are no $3\,{\rightarrow }\,2$ moves or there is a $3\,{\rightarrow }\,2$ move that is not the start of a geodesic to ${\mathcal T}_0$. This idea seems worth exploring, but we leave it for others to pursue.

## Modifying Triangulations with Arcs

Part (a) of the input data produced the layered filled triangulation ${\mathcal T}$ of Sect. 3, which comes enriched with a barycentric link *L*. Part (b) of the input data is a sequence of Pachner moves $(P_i)$ converting ${\mathcal T}$ to the base triangulation ${\mathcal T}_0$ described in Sect. 7. The next step of our algorithm is to apply the moves $(P_i)$ to ${\mathcal T}$, carrying the link *L* along as we go.

### Pachner Moves with Arcs

In this section, we describe how we keep track of the barycentric arcs as Pachner moves are performed. Throughout this section, ${\mathcal T}$ is a triangulation with barycentric arcs and *P* is a Pachner move transforming ${\mathcal T}$ into the triangulation ${\mathcal S}=P{\mathcal T}$.

**Fig. 7:**
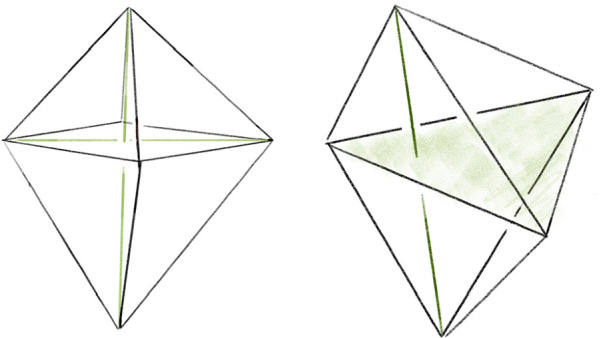
Two bipyramids with superimposed triangulations corresponding to before and after applying the $4\,{\rightarrow }\,4$ move and $2\,{\rightarrow }\,3$ or $3\,{\rightarrow }\,2$ moves

**Fig. 8:**
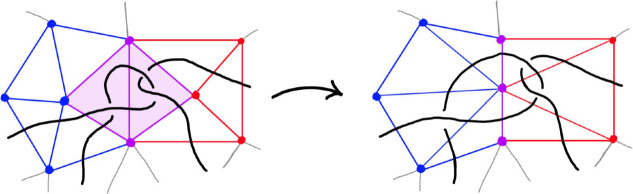
Cartoon showing the difficulty of doing a $2\,{\rightarrow }\,0$ move with arcs present. At left, the two tetrahedra in the pillow to be collapsed are shaded. Here, you should regard the vertical purple arc as the valence-2 edge, with the blue and red dots opposite being cross-sections of the two edges of the pillow that become identified in the collapse. The problem is that we have to push all the topology of the *link* out of the pillow before we collapse it, requiring us to move arcs into many of the tetrahedra adjacent to the pillow

Recall that the *simple Pachner moves* are $2\,{\rightarrow }\,3$, $3\,{\rightarrow }\,2$, and $4\,{\rightarrow }\,4$. Each simple Pachner move *P* takes a triangulated ball *B* in ${\mathcal T}$, possibly with boundary faces glued together, and re-triangulates *B* without changing the triangulation of $\partial B$ to obtain ${\mathcal S}$. The arcs of the link *L* contained in the ball are initially encoded using the barycentric coordinates of ${\mathcal T}$, and we need to re-express these arcs in the new barycentric coordinate system of ${\mathcal S}$. We model each simple Pachner move as a pair of triangulations of concrete bipyramids in ${\mathbb R}^3$, as shown in Fig. 7. We identify the tetrahedra in ${\mathcal T}$ and ${\mathcal S}$ involved in *P* with tetrahedra in the corresponding bipyramid in ${\mathbb R}^3$. This identification allows us to map barycentric arcs from ${\mathcal T}$ into ${\mathbb R}^3$, and then to map these arcs in ${\mathbb R}^3$ into ${\mathcal S}$. The remainder of Sect. 5 details how this is used to give a method $\mathtt{with\_arcs}[P]$ that applies a simple Pachner move *P* to ${\mathcal T}$ while transferring the barycentric arcs from ${\mathcal T}$ to ${\mathcal S}$. This approach cannot work for the $2\,{\rightarrow }\,0$ move, as demonstrated by Fig. 8. To implement $\mathtt{with\_arcs}[2\,{\rightarrow }\,0]$, we factor the $2\,{\rightarrow }\,0$ move into a sequence of $2\,{\rightarrow }\,3$ and $3\,{\rightarrow }\,2$ moves as described in Sect. 6.

### Weak Barycentric Arcs

It will be useful to have a mild generalization of barycentric arcs that allows for negative barycentric coordinates. Identify ${\mathbb R}^3$ with the hyperplane $\sum _ix_i=1$ in ${\mathbb R}^4$. The barycentric coordinates $(x_1,x_2,x_3,x_4)$ associated to the vertices of the standard simplex in ${\mathbb R}^4$ extend to all of ${\mathbb R}^3$. A *weak barycentric coordinate* is a tuple $(x_1,x_2,x_3,x_4)$ describing a point in this hyperplane. Identifying a tetrahedron $\tau$ in ${\mathcal T}$ with the standard 3-simplex in ${\mathbb R}^4$, a *weak barycentric point* in $\tau$ is a weak barycentric coordinate defining a point in the hyperplane $\sum _ix_i=1$. A *weak barycentric arc* *a* is a pair of weak barycentric points (*u*, *v*) associated to a tetrahedron $\tau$.

Note that a weak barycentric arc does not generally define a geometric object in the triangulation ${\mathcal T}$. However, a weak barycentric arc may contain a sub-arc that is a genuine barycentric arc. From our identification of $\tau$ with the standard 3-simplex in ${\mathbb R}^4$, the intersection of a weak barycentric arc with the standard simplex is a possibly empty barycentric arc. As ultimately we only want to work with barycentric arcs, we use a trimming procedure that takes a weak barycentric arc *a* in $\tau$ and replaces it with the maximal barycentric arc it contains.

### Putting the Problem into ${\mathbb R}^3$

We model the simple Pachner moves as a pair of triangulations of concrete bipyramids in ${\mathbb R}^3$. We can identify the tetrahedra in ${\mathcal T}$ and ${\mathcal S}$ involved in the move with tetrahedra in the corresponding bipyramid. This identification allows us to map barycentric arcs from ${\mathcal T}$ into ${\mathbb R}^3$ and then to map these arcs in ${\mathbb R}^3$ back to ${\mathcal S}$. Combining these processes transfers arcs from ${\mathcal T}$ to ${\mathcal S}$. We explain this in detail for the $2\,{\rightarrow }\,3$ move.

Recall that the $2\,{\rightarrow }\,3$ move takes two tetrahedra glued along a face *F*, deletes the face *F*, adds a vertical edge, and re-triangulates so that there are three tetrahedra assembled around the new vertical edge; see Fig. 4. The bipyramid is built from a pair of tetrahedra $\Delta _N$ and $\Delta _S$ in ${\mathbb R}^3$ sharing a single face. Let $A=(3,0,0)$, $B=(0,0,3)$, and $C=(0,3,0)$ be the corners of the common face and let $N=(0,0,0)$ and $S=(2,2,2)$ be corners of the bipyramid lying over and under the common face. These particular points are chosen as they are symmetric with respect to affine maps. There is also a triangulation of this bipyramid consisting of three tetrahedra assembled around a central edge running from *N* to *S*. We label these tetrahedra $\Delta _A,\Delta _B,\Delta _C$, where $\Delta _A$ is the tetrahedron excluding *A*, and likewise for *B* and *C*. The $2\,{\rightarrow }\,3$ move replaces the $2$-tetrahedron triangulation with this $3$-tetrahedron triangulation.

Let $\tau _N$ and $\tau _S$ be tetrahedra in ${\mathcal T}$ sharing a face *F*. To do the $2\,{\rightarrow }\,3$ move determined by the face *F* while preserving the barycentric arc data, we map the arcs in $\tau _N$ and $\tau _S$ into the model bipyramid in ${\mathbb R}^3$ using the barycentric coordinates. We first require a fixed map from the vertices of $\tau _N$ and $\tau _S$ to the bipyramid of ${\mathbb R}^3$. The particular assignment is determined by whether the move is called from the point of view of $\tau _N$ or $\tau _S$ and from the internal labeling of the vertices of the face *F*. We use a map identifying $\tau _N$ with $\Delta _N$ and $\tau _S$ with $\Delta _S$. We also require a map identifying the three new tetrahedra in ${\mathcal S}$ with the tetrahedra $\Delta _A,\Delta _B,\Delta _C$ in the other triangulation of the bipyramid. In ${\mathcal S}$, let $\sigma _A$, $\sigma _B$, and $\sigma _C$ be the three tetrahedra incident the new central edge. For precise details on these vertex correspondences, see the file barycentric_geometry.py in [20].

Barycentric coordinates in $(\tau _N,\tau _S)$ and $(\sigma _A,\sigma _B,\sigma _C)$ determine unique points in the bipyramid via the vertex correspondence and the barycentric coordinates of the two triangulations of the bipyramid. This then determines a map sending barycentric arcs from ${\mathcal T}$ and ${\mathcal S}$ to arcs in the bipyramid. If *a* is a barycentric arc in a tetrahedron $\tau$ in ${\mathcal T}$ or ${\mathcal S}$, let $\mathtt{{arc\_embedding}}_{\tau }(a)$ be the corresponding arc in the bipyramid.

#### Remark 5.1

It is crucial that we check if the arcs in the bipyramid are in general position (in the sense of Sect. 2.2) with the $3$-tetrahedron triangulation. For example, it can happen that an arc that is disjoint from the 1-skeleton of the $2$-tetrahedron triangulation of the bipyramid intersects the vertical edge in the $3$-tetrahedron triangulation. When there is any such *general position failure*, we perturb the north and south poles of the bipyramid slightly and repeat the above process.

PL arcs in the bipyramid can also be described by barycentric arcs in ${\mathcal T}$ and ${\mathcal S}$. Given distinct points *p* and *q* in the bipyramid, there is a sequence of barycentric arcs $a_j$ contained in either $(\tau _N,\tau _S)$ or in $(\sigma _0,\sigma _1,\sigma _2)$, such that the line segment between *p* and *q* is the concatenation of the line segments $\mathtt{{arc\_embedding}}_{a_j.\mathtt{{tet}}}(a_j)$, where $a_j.\texttt{tet}$ is the tetrahedron containing $a_j$. This is done by describing the line segment from *p* to *q* as a weak barycentric arc $a_{p,q}(\tau )$ for each tetrahedron $\tau$ in the relevant triangulation, then trimming these weak barycentric arcs. The function that takes an arc *a* in the bipyramid and adds this sequence of barycentric arcs to the tetrahedra $(\tau _N,\tau _S)$ or $(\sigma _A,\sigma _B,\sigma _C)$ in ${\mathcal T}$ or ${\mathcal S}$ is denoted $\mathtt{{arc\_pullback}}_{{\mathcal T}}(a)$ or $\mathtt{{arc\_pullback}}_{{\mathcal S}}(a)$.

### Transferring the Arcs

By combining $\mathtt{{arc\_pullback}}$ and $\mathtt{{arc\_embedding}}$, we define a function $\mathtt transfer\_arcs$ that takes barycentric arcs in ${\mathcal T}$, maps them into the bipyramid, then pulls them back to ${\mathcal S}$. This enables us to build a version of the $2\,{\rightarrow }\,3$ move that transfers arcs from ${\mathcal T}$ to ${\mathcal S}$:

#### Algorithm 5.2

$$\mathtt{with\_arcs}[2\,{\rightarrow }\,3]$$

1. Identify $\tau _N$ with $\Delta _N$ and $\tau _S$ with $\Delta _S$ in the bipyramid in ${\mathbb R}^3$.
2. Do the $2\,{\rightarrow }\,3$ move to get new tetrahedra in ${\mathcal S}$ that are identified with the corresponding tetrahedra in the $3$-tetrahedron triangulation of the bipyramid.
3. Apply $\mathtt {transfer\_arcs}$: for each arc *a* in $\tau _N$ and $\tau _S$, append $\mathtt{{arc\_embedding}}_{a.\mathtt tet}(a)$ to the list $\texttt{arcs}$. For each arc *b* in $\texttt{arcs}$, apply $\mathtt{{arc\_pullback}}_{{\mathcal S}}(b)$.

The above approach is easily modified to accommodate the $3\,{\rightarrow }\,2$ move. For the $4\,{\rightarrow }\,4$ move, this approach works with minor modifications if one uses a bipyramid with square base. We therefore can can implement methods $\texttt {with\_arcs}[3\,{\rightarrow }\,2]$ and $\texttt {with\_arcs}[4\,{\rightarrow }\,4]$ that handle barycentric arcs.

### Simplifying Arcs

**Fig. 9:**
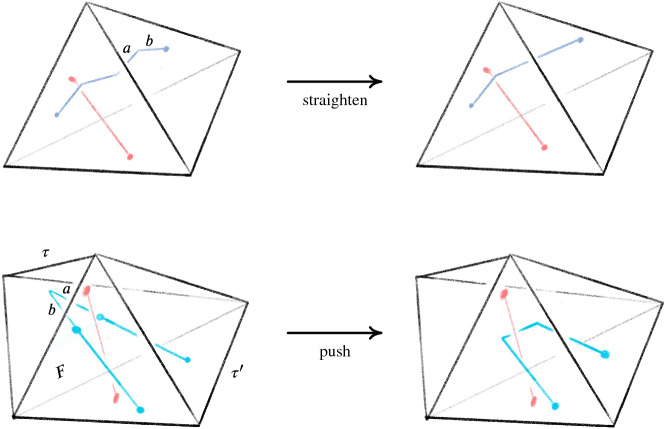
The *straighten* move removes unnecessary bends in the link, and the *push* move reduces unnecessary intersections with the 2-skeleton

Given the inputs (a) and (b) of Sect. 1.2, the Pachner moves with arcs machinery always produces the desired link *L* in the base triangulation ${\mathcal T}_0$. However, even in the smallest examples, applying the sequence of Pachner moves to ${\mathcal T}$ produces incredibly complicated configurations of arcs in ${\mathcal T}_0$ encoding *L*. This complexity makes necessary computational geometry tasks prohibitively expensive. Fortunately, much of this complexity is not topologically essential, and the number of arcs can be decreased dramatically by the basic simplifications we now describe. Without these, applying our full algorithm to an ideal triangulation $\mathring{{\mathcal T}}$ with just two tetrahedra resulted in 838 arcs and an initial link diagram with 5130 crossings; with the simplifications, there are 19 arcs and 35 crossings. A 3-tetrahedra ideal triangulation resulted in 129, 265 arcs compared to 27 with simplifications. An example with ten tetrahedra would be impossible without these simplifications. Our two kinds of simplification moves are shown in Fig. 9 .

The first is straighten, which takes as input a tetrahedron $\tau$ with barycentric arcs. It then checks for each arc *a* in $\tau$ if the pair of arcs *a* and $b=a.\texttt{next}$ can be replaced with a single arc that runs from $a.\texttt{start}$ and $b.\texttt{end}$. The check is that no other arc in $\tau$ has an interior intersection with the triangle spanned by *a* and *b*. The other move is push, which removes unnecessary intersections with ${\mathcal T}^2$. When *a* starts on the same face *F* that $b=a.\texttt{next}$ ends on, it checks whether any other arc intersects the triangle *a* and *b* span. If there are none, the move replaces *a* and *b* with an arc in the tetrahedron $\tau '$ glued to $\tau$ along *F*. This often produces a bend that can then be removed by a straighten move.

### Putting the Pieces Together

Let ${\mathcal T}$ be a layered filling triangulation with arcs encoding the core curves of the filling and let $(P_i)$ be simple Pachner moves reducing ${\mathcal T}$ to ${\mathcal T}_0$. By factoring any $2\,{\rightarrow }\,0$ or $0\,{\rightarrow }\,2$ moves, see Sect. 6, we can always turn a sequence of Pachner moves into a sequence of simple Pachner moves. Our process for producing a barycentric link in ${\mathcal T}_0$ that is isotopic to the initial *L* is:

#### Algorithm 5.3

$$\mathtt{with\_arcs[apply\_Pachner\_moves]}({\mathcal T},(P_i))$$

Start with ${\mathcal T}':={\mathcal T}$ and loop over the $P_1,P_2,\dots ,P_n$ as follows:

1. Apply $\mathtt {with\_arcs}[P_i]$ to ${\mathcal T}'$ to get $P_i{\mathcal T}'$ with arcs representing *L*. Set ${\mathcal T}':=P_i{\mathcal T}'$.
2. Loop over the tetrahedra $\tau$ in ${\mathcal T}'$, applying $\texttt{push}$ and $\texttt{straighten}$ until the arcs stabilize.

### Computational Geometry Issues

Our algorithm requires many geometric computations with barycentric arcs, e.g. to test for one of our simplifying moves and to ensure we do not violate the general position requirement of Sect. 2.2. Difficult and subtle issues can arise here, and much work has been done to ameliorate them; see [59] for a survey. We took the approach of having all coordinates in ${\mathbb Q}$ so that we can do these computations exactly. This entails a stiff speed penalty and leads to points represented by rational numbers with overwhelmingly large denominators.

We handle such denominators by rounding coordinates at each stage. As long as the rounding process does not move the endpoint of an arc more than one-fourth the minimum distance between any pair of arcs in the link, the isotopy class of the link is unchanged by rounding, see [64, p. 316]. We therefore guarantee correctness by computing the minimum distance between arcs at every step of the algorithm and then varying the rounding precision accordingly.

Alternatively, when the input manifold is hyperbolic, one can generally efficiently certify correctness of the output diagram after the fact by checking that its exterior is homeomorphic to the manifold in part (a) of the input. This is only marginally faster than dynamically varying the rounding precision in our current implementation, so the final version only uses the approach that is independent of hyperbolicity. However, this does give us a completely independent check on the correctness of our code.

## Factoring the 2-to-0 Move

**Fig. 10:**
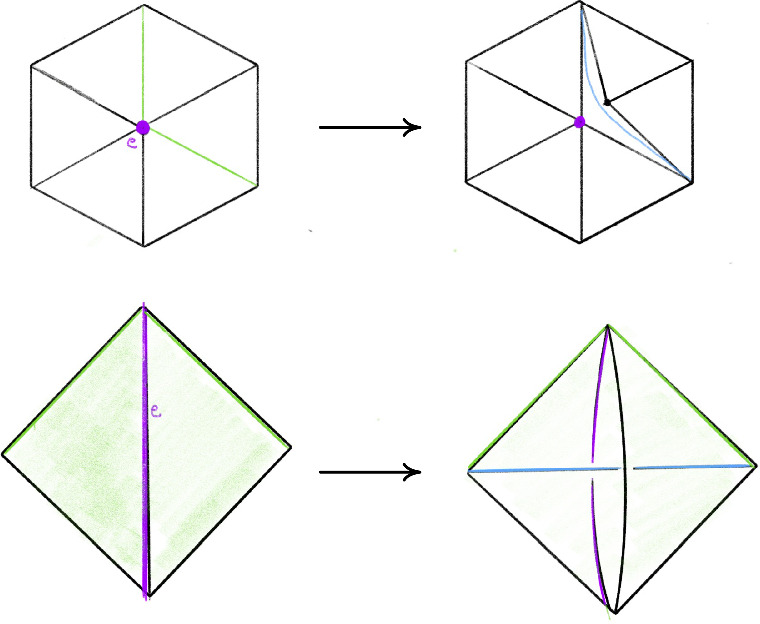
At top, a cross section of a $0\,{\rightarrow }\,2$ move; at bottom is a close-up of the inflation of the pillow. The move is performed on the pair of green faces meeting along the purple edge *e* at left. The resulting pillow is a *bird beak*, which splits open the book of tetrahedra about *e*. In the top right, the purple and black dots give edges that join together above and below the cross section

As mentioned in Sect. 5.1, we factor each $2\,{\rightarrow }\,0$ move into a sequence of $2\,{\rightarrow }\,3$ and $3\,{\rightarrow }\,2$ moves so that we can carry along the barycentric link. This factorization is quite delicate in certain unavoidable corner cases; we next outline our method and then provide a detailed account in Sect. 6.1. To begin to understand the $2\,{\rightarrow }\,0$ move, first consider its inverse $0\,{\rightarrow }\,2$ move shown in Fig. 10. The possible $0\,{\rightarrow }\,2$ moves in Fig. 10 correspond to a pillow splitting open the *book of tetrahedra* around the edge *e*. Following [63], we call this pillow a *bird beak* with upper and lower mandibles that pivot around the two outside edges of the beak (viewed from above, these are the purple and black vertices in the top right of Fig. 10). On both sides of the bird beak are *half-books* of tetrahedra, together forming a *split-book*. When applying the inverse $2\,{\rightarrow }\,0$ move, the two half-books combine to form a book of tetrahedra assembled around the central edge.

**Fig. 11:**
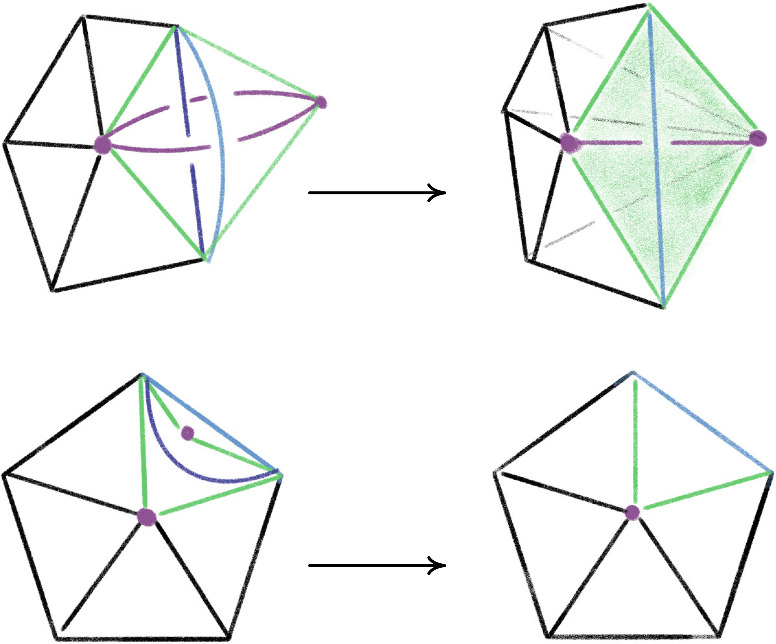
The base case of the $2\,{\rightarrow }\,0$ move at top with the cross section at bottom

The simplest $2\,{\rightarrow }\,0$ move is when there are two valence-2 edges that are opposite each other on a single tetrahedron, as shown in Fig. 11; equivalently, one of the half-books has a single tetrahedron. This *base case* is handled by Matveev’s *V* move, the composition of four $2\,{\rightarrow }\,3$ and $3\,{\rightarrow }\,2$ moves of [46, Fig. 1.15]. To reduce other instances of the $2\,{\rightarrow }\,0$ move to the base case, we rotate a mandible of the bird beak, moving tetrahedra from one half-book to the other until one contains only a single tetrahedron. Because the tetrahedra in the split-book may repeat or be glued together in strange ways, this is rather delicate. When things are sufficiently embedded, Segerman [63] showed:

### Proposition 6.1

Suppose *e* is a valence-2 edge where the half-books adjacent to the bird beak are embedded and contain *m* and *n* tetrahedra respectively. Then the $2\,{\rightarrow }\,0$ move can be implemented by at most $2\cdot \min (m,n)+2$ basic $2\rightarrow 3$ and $3\rightarrow 2$ moves.

### Proof

We can rotate a mandible by one tetrahedron using the two basic moves of [63, Fig. 11]. With $\min (m,n)-1$ such rotations we can reduce the smaller of the half-books to a single tetrahedron. As already noted, the base case can be done in four moves. $\square$

### Remark 6.2

One cannot in general factor a $2\,{\rightarrow }\,0$ move into a sublinear number of $2\,{\rightarrow }\,3$ and $3\,{\rightarrow }\,2$ moves: the $2\,{\rightarrow }\,0$ move amalgamates two edges of valence $m+1$ and $n+1$ into a single edge of valence $m+n$, and each $2\,{\rightarrow }\,3$ or $3\,{\rightarrow }\,2$ move only changes valences by a total of 12 (counting with multiplicity).

**Fig. 12:**
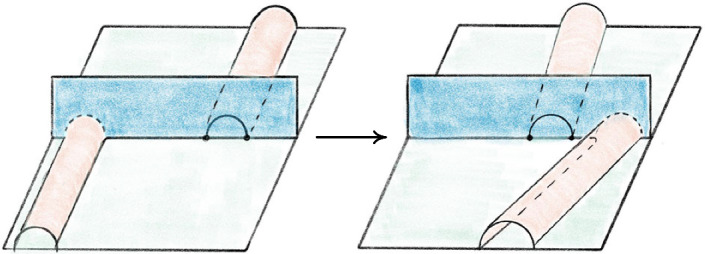
The *endpoint-through-endpoint* move in a special spine

The tricky case is when additional faces of the bird beak are glued to each other. There are two fundamentally different ways for this to happen, shown in Figs. 18 and 19 of Sect. 6.1. The untwisting of these extremely confusing arrangements is done by the endpoint-through-endpoint move of Fig. 12, which is in the dual language of special spines from Sect. 6.1. Matveev’s factorization of the endpoint-through-endpoint move is described in [46, Fig. 1.19]. We simplify this factorization from 14 moves to 6; the key is Proposition 6.4. This simplification was essential for determining the exact sequence of moves needed to factor the $2\,{\rightarrow }\,0$ move. Dual to the endpoint-through-endpoint move are a pair of *untwist the beak* moves, one for each of the situations in Figs. 18 and 19, see Sect. 6.1. We can thus factorize the $2\,{\rightarrow }\,0$ move as follows:

### Algorithm 6.3

$$\texttt{factor}[2\,{\rightarrow }\,0]$$

1. If we are in the base case, do the sequence of moves in the triangulation dual to the factorization of the *V* move in [46, Fig. 1.15] and exit.
2. If we are in the twisted cases described by Figs. 18 and 19 in Sect. 6.1, do the appropriate untwist the beak move. Otherwise, rotate the mandible by one tetrahedron.
3. Go to to step 1.

**Fig. 13:**
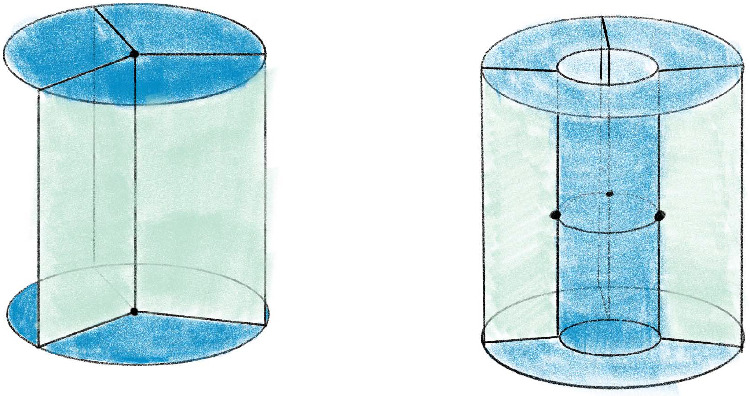
The *T* move in the special spine dual to the $2\,{\rightarrow }\,3$ move

**Fig. 14:**
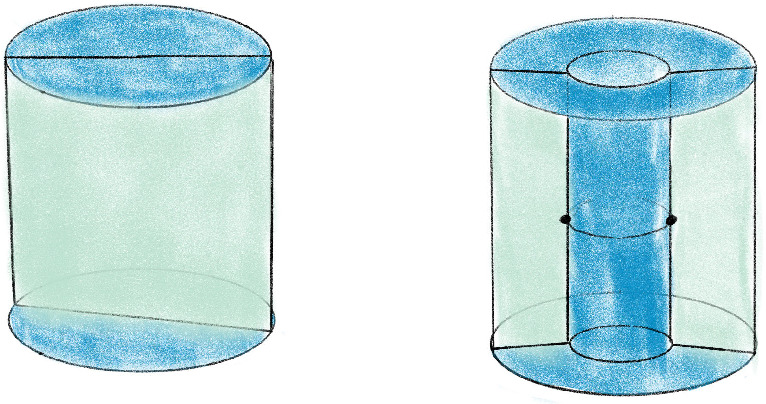
The lune move *L* in the special spine dual to the $0\,{\rightarrow }\,2$ move

**Fig. 15:**
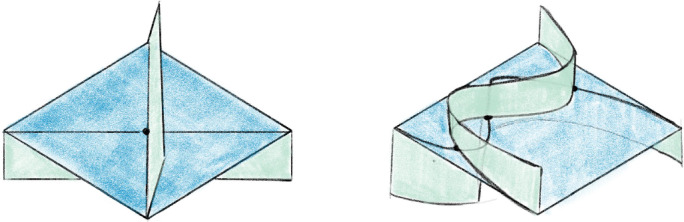
Matveev’s *V* move

### Dealing with Twisted Beaks Using Special Spines

In this section, we detail how to handle the situation where additional faces of the bird beak are identified, which can happen because the tetrahedra in the split-book, shown in the upper-right of Fig. 10, may repeat or be glued together in strange ways. To handle this delicate issue, we need to take the dual viewpoint.

Dual to a triangulation is a *special spine*. A *spine* of a compact 3-manifold *M* with nonempty boundary is a polyhedron *P* such that *M* collapses to *P*. A spine of a closed 3-manifold *M* is a spine of the complement of an open ball in *M*. Any triangulation ${\mathcal T}$ of *M* determines a dual cellulation ${\mathcal S}$ whose 2-skeleton is a spine, see [46, Fig. 1.5]; the class of such spines are the *special spines*,

The Pachner moves correspond to dual moves in the special spine. Generally, it is easier to reason about the dual moves modifying special spines, as there is a type of graphical calculus. The move on a special spine dual to the $2\,{\rightarrow }\,3$ is called the *T* move and its inverse dual to the $3\,{\rightarrow }\,2$ move is denoted $T^{-1}$, see Fig. 13. The special spine move corresponding to the $0\,{\rightarrow }\,2$ move is the *lune* move shown in Fig. 14, and is denoted *L*. The move dual to $2\,{\rightarrow }\,0$ is the inverse lune move $L^{-1}$. It is also useful to have a certain compound move, Matveev’s *V* move, shown in Fig. 15, which is a special case of the lune move that can be realized as a sequence of four *T* and $T^{-1}$ moves [46, Fig. 1.15]. Matveev showed that generally the $0\,{\rightarrow }\,2$ and $2\,{\rightarrow }\,0$ moves can be factored as a sequence of $2\,{\rightarrow }\,3$ and $3\,{\rightarrow }\,2$ moves. In this section, we describe a simplification of one part of Matveev’s factorization, reducing the number of moves necessary for that part from 14 to 6.

**Fig. 16:**
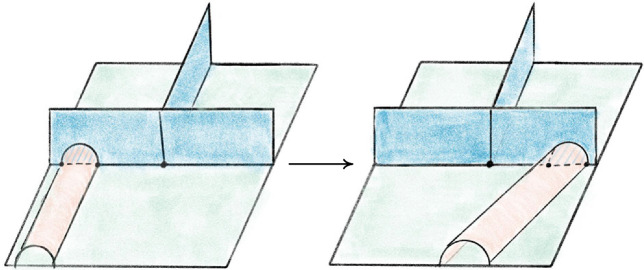
The *endpoint-through-vertex* move on a special spine. There are three vertices of the singular locus in both configurations. This is dual to the move rotating a mandible

The first move we need to transfer tetrahedra from one half-book to the other is the dual version of the endpoint-through-vertex move in Fig. 16. The endpoint-through-vertex move involves a single *T* and $T^{-1}$ move. When the split-book is not twisted in strange ways, this move suffices to reduce to the base case. A thorough account of this move, describing both the triangulation and special spine versions, is given in [63].

**Fig. 17:**
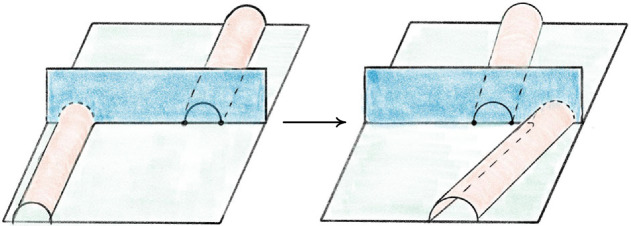
The *endpoint-through-endpoint* move in a special spine. There are only two vertices of the singular locus in each picture. You can walk through the “tunnel” starting in the lower left and end up “behind” the vertical blue wall; in contrast, the back tunnel dead-ends into the vertical blue wall. This move is needed to reduce to the base case when the tetrahedra in the split-book are twisted up in various ways. We implement this move for the dual triangulation as a sequence of six $2\,{\rightarrow }\,3$ and $3\,{\rightarrow }\,2$ moves

The special spine move needed to deal with twisted half-books is the endpoint-through-endpoint move pictured in Fig. 17. A factorization of the endpoint-through-endpoint move is given in [46, Fig. 1.19], and it is this factorization that we simplify from 14 to 6 moves. Converting from the spine version of the endpoint-through-endpoint move to the triangulation version is quite subtle, so reducing the number of moves in the factorization is incredibly helpful.

Our simplification of the factorization is in fact a simplification of one part of the endpoint-through-endpoint move. Specifically, the factorization in [46, Fig. 1.19] consists of three stages. The first stage uses the move sequence $V,T,T^{-1}$, the second the sequence $T,T^{-1}$, and the final stage is, up to taking the mirror image, the inverse of the first stage. Expanding *V* to its four constituent *T* and $T^{-1}$ moves means both the first and third stages require six *T* and $T^{-1}$ moves each. However, the next proposition shows that one only needs two *T* moves for each of these stages, saving us eight moves overall:

#### Proposition 6.4

The below move on a special spine can be achieved by two *T* moves:
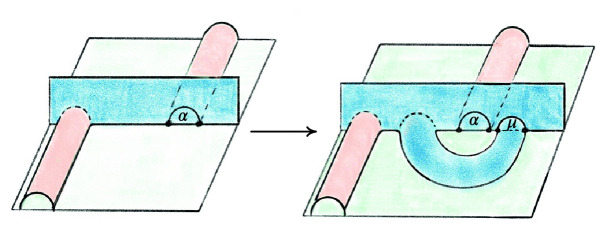


There are two vertices initially, and four at the end. You can enter any of the lower tunnels from the top-left green region; for the U-tunnel at right you would reach a dead-end at the vertical blue wall at $\mu$.

#### Proof

Zooming in on the part of the figure near the vertices, we see:
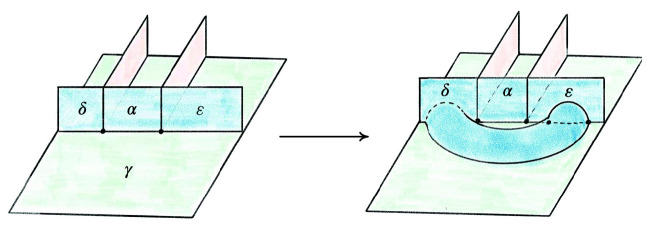


We can then realize the move as follows:

1. Rotate the walls left of $\alpha$:
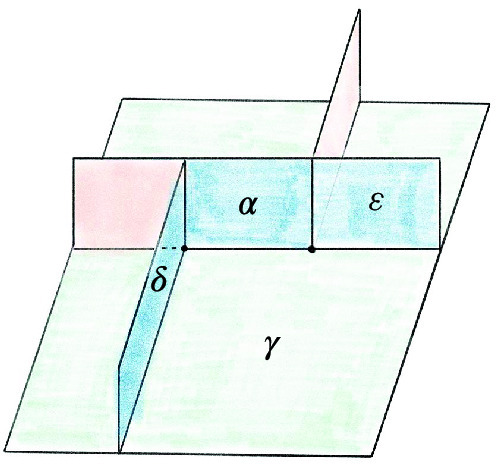
2. Fold so $\alpha$, $\varepsilon$, and $\gamma$ are in the same plane:
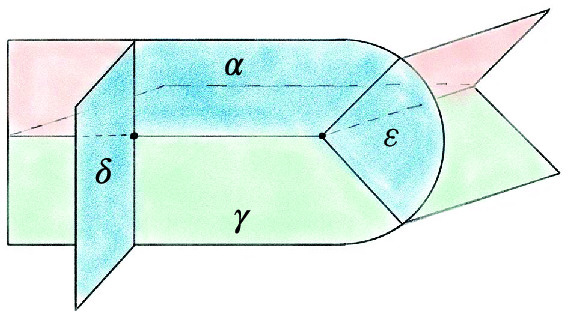
3. Follow [46, Fig. 1.15] to obtain:
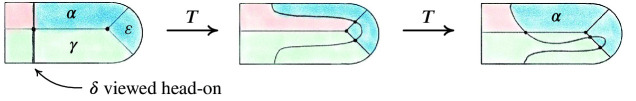
4. Redraw as:
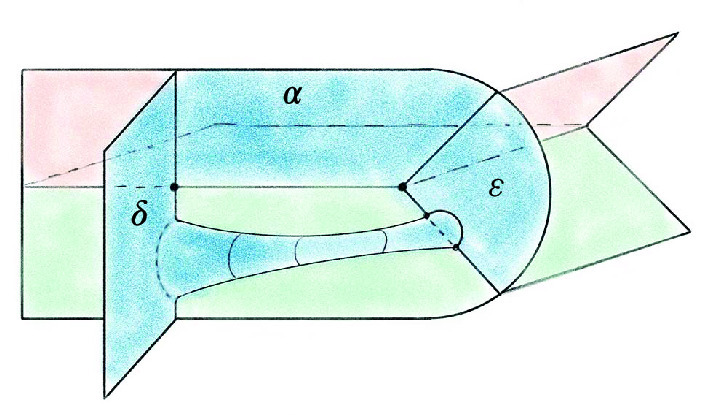
5. Reverse the folds to obtain:
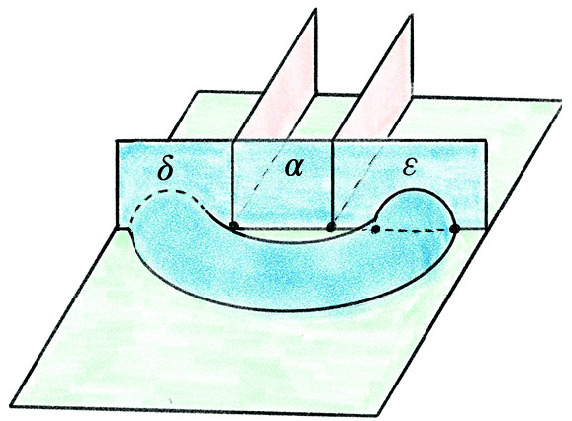

This completes the proof. $\square$

#### Corollary 6.5

The endpoint-through-endpoint move can be completed as a sequence of six moves using the moves *T* and $T^{-1}$ dual to the $2\,{\rightarrow }\,3$ and $3\,{\rightarrow }\,2$ moves.

#### Proof

Following [46, Fig. 1.19], the endpoint-through-endpoint move is obtained doing the move in Proposition 6.4, then doing one *T* and one $T^{-1}$ move, and ending with the inverse of Proposition 6.4. $\square$

We now need to dualize the endpoint-through-endpoint move to the triangulation setting. We call the dual version of the endpoint-through-endpoint move the *untwist the beak* move. Suppose we have a bird beak in a split-book of tetrahedra. There are two possible twisted complexes contained in a half-book that can arise, preventing us from rotating the mandible using the dual endpoint-through-vertex move. Both of these are overcome using the untwist the beak move.

**Fig. 18:**
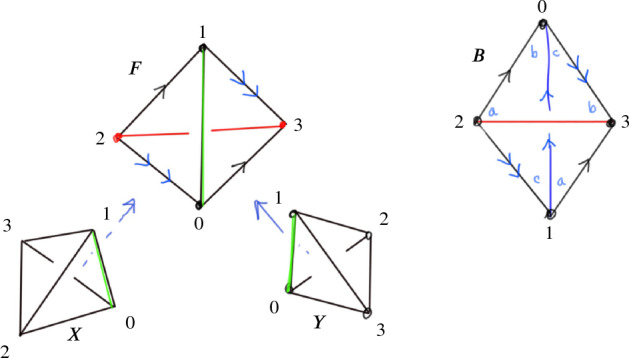
One of the possible twisted complexes in a split-book of tetrahedra. Here, the tetrahedron *F* sits in front of *B* forming the valence-2 edge; said edge connects vertices 2 and 3 in both tetrahedra, with the pair of opposite edges joining vertices 0 and 1 forming the “equator” around it. The back tetrahedron *B* forms a solid torus with its back two faces glued as indicated by the labels $\{a,b,c\}$. The face opposite vertex 2 in tetrahedron *X* is glued to the face of *F* opposite vertex 3 so that the two edges connecting vertices 0 and 1 coincide; the tetrahedron *Y* is glued to *F* analogously

In the first twisted case, there are at least two distinct tetrahedra glued to the front of the pillow, and the back of the pillow is glued to itself as indicated in Fig. 18, producing a solid torus. In this case, the half-book around the front edge and the half-book around the back edge overlap. In Fig. 18, the leftmost and rightmost tetrahedra in the front half-book, labeled *X* and *Y*, appear in both the front and back half-books.

**Fig. 19:**
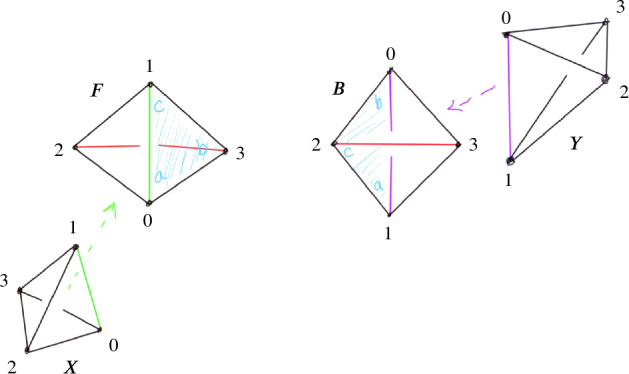
Another possible twisted complex in a split-book of tetrahedra. The tetrahedron *F* sits in front of *B* forming the valence-2 edge as in Fig. 18. These tetrahedra share a third pair of faces (shaded with corners labeled $\{a,b,c\}$) which must be offset as shown as otherwise the $2\,{\rightarrow }\,0$ move would change the topology. Here, the back face of *X* is glued to the unshaded front face of *F* so that the vertices 0 and 1 coincide; similarly, the front face of *Y* is glued to unshaded back face of *B* so that the vertices 0 and 1 coincide

The other possible twisted case involves a front face of the pillow being glued to the back face of the pillow, as indicated in Fig. 19. In this case, the front tetrahedron of the pillow is part of the half-book around the back edge, and the back tetrahedron of the pillow is part of the half-book around the front edge.

For both possible twisted cases, the particular sequence of $2\,{\rightarrow }\,3$ and $3\,{\rightarrow }\,2$ moves corresponding to factorization of the endpoint-through-endpoint move in Corollary 6.5 was found by searching possible sequences, using the valences of various edges as a guide. More details on the sequence can be found in the implementation, see the file mcomplex_with_expansion.py in [20]. The factorization of the $2\,{\rightarrow }\,0$ described at the end of Sect. 6 then follows.

## Building the Initial Diagram

**Fig. 20:**
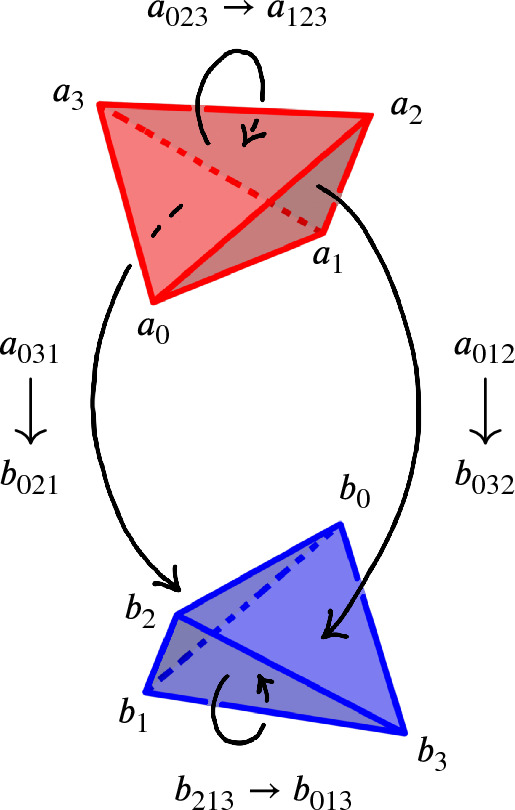
The base triangulation ${\mathcal T}_0$ in ${\mathbb R}^3$

The base triangulation ${\mathcal T}_0$ of $S^3$ has two tetrahedra and one vertex and is shown in Fig. 20; its isomorphism signature in the sense of [10, Sect. 3.2], which completely determines the triangulation, is cMcabbgdv. We next give the method for obtaining a planar diagram *D* for a barycentric link *L* in ${\mathcal T}_0$. We first build a PL link in ${\mathbb R}^3$ representing *L* and then project it onto a plane to get *D*.

**Fig. 21:**
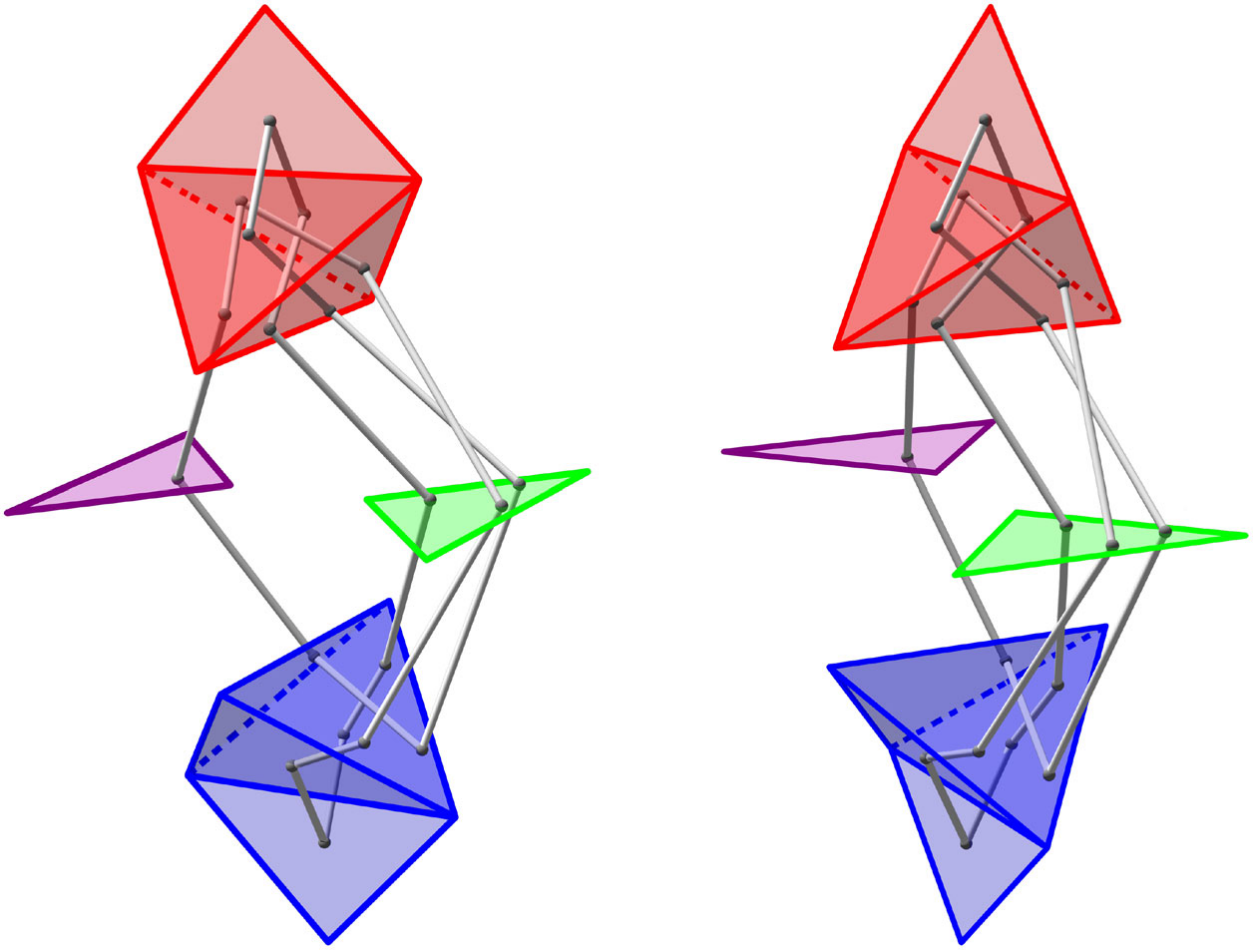
Two views of the same link realized by the base triangulation ${\mathcal T}_0$ using the fins and lenses shown

We cut open ${\mathcal T}_0$ along its faces and embed the resulting pair of tetrahedra in ${\mathbb R}^3$ as shown in Fig. 20. This cuts open the link *L* along its intersections with the faces of ${\mathcal T}_0$, resulting in a collection of curves in ${\mathbb R}^3$ inside the two tetrahedra. To reconnect these curves and recover *L*, we use *fins* and *lenses* as shown in Fig. 21 to interpolate between pairs of faces that are identified in ${\mathcal T}_0$. There are two triangular fins, one attached vertically to each tetrahedron, with each fin corresponding to one of the two valence-1 edges of ${\mathcal T}_0$. The gluing of two faces incident to a valence-1 edge is realized by folding them onto the corresponding fin. Thus for each barycentric arc that ends in a face corresponding to a fin, we add the line segment joining this endpoint of the arc to the corresponding point in the fin.

The two triangular lenses lie between the two tetrahedra in a horizontal plane. There is an affine map taking the corresponding face in the top tetrahedron to its lens and a second affine map taking the lens to the corresponding face in the bottom tetrahedron, arranged so their composition is the face pairing in ${\mathcal T}_0$. For every arc in the top tetrahedron ending on a face corresponding to a lens, we add the line segment between the endpoint and its image under the affine map to the lens. For each such segment that terminates on a lens, we add the line segment from this endpoint to its image in the face of the bottom tetrahedron under the affine map. This results in a PL link in ${\mathbb R}^3\subset S^3$ that must be isotopic to *L*: just imagine puffing out the two tetrahedra to fill all of $S^3$ following the guides given by the fins and lenses.

Given a collection of line segments in ${\mathbb R}^3$ corresponding to the link *L*, we can build a diagram for *L* by projecting the line segments onto a plane, computing the crossing information, and assembling this into a planar diagram. Our default choice is roughly to project onto the plane of the page in Fig. 21, with the (so far unused) fall-back of a small random matrix in $\textrm{ SL}_{3} {\mathbb Z}$ if a general-position failure occurs. The link diagrams resulting from this process have many more crossings than is necessary, and we deal with this in Sect. 8. Still, the specific configuration of fins, lenses, and projection was chosen to try minimize the number of crossings created at this stage; our initial approach used a more compact embedding where the tetrahedra shared a face, and this produced much larger diagrams.

## Simplifying Link Diagrams

**Fig. 22:**
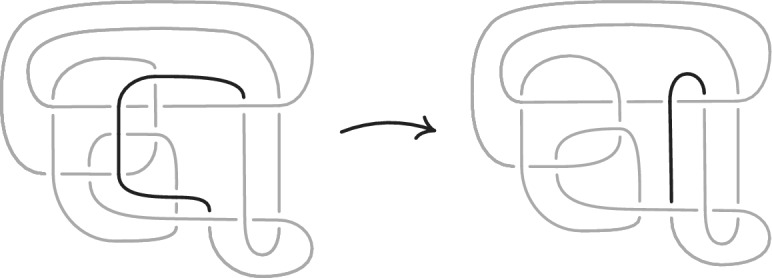
An example of the strand pickup method for diagram simplification. At left, an *overstrand*, which runs over each crossing it participates in, is indicated by the darker line. At right is the result of isotoping the verstrand, fixing its endpoints, to get a diagram with fewer crossings. The best possible location for an overstrand can be found by solving a weighted shortest-path problem in the planar dual graph to the original diagram

**Fig. 23:**
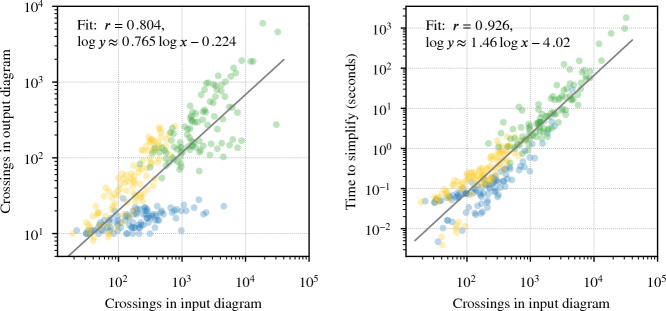
Simplifying 300 diagrams with between 19 and 32, 095 crossings, drawn from Sects. 10 and 11.3. The dramatic amount of simplification is shown at left, with an *n*-crossing knot turned into one with $O(n^{0.8})$ crossings. The running time shown on the right is roughly $O(n^{1.5})$

We now sketch how we simplified the initial link diagram constructed in Sect. 7, which sometimes had 10, 000–100, 000 crossings, to produce the final output of our algorithm for Find Diagram. Previous computational work focused on simplifying diagrams with 20 or fewer crossings [12, 33]. In that regime, random Reidemeister moves combined with flypes are extremely effective in reducing the number of crossings. However, these techniques alone proved inadequate for our much larger links. Instead, we used the more global *strand pickup* method of Fig. 22. This technique was introduced by the second author and included in SnapPy [18] since version 2.3 (2015), but not previously documented in the literature. It has similarities with the arc representation/grid diagram approach of [22–24], but it works with arbitrary planar diagrams. When applying the pickup move, we start with the longest overstrands and work towards the shorter ones if no improvement is made. When a pickup move succeeds, we do more basic simplifications before looking for another pickup move. We also do the same move on understrands, going back and forth between the two sides until the diagram stabilizes; for details, see [51]. The high amount of simplification and sub-quadratic running time are shown in Fig. 23. As further evidence of its utility, we note that it strictly monotonically reduces the unknot diagrams $D_{28}$, $D_{43}$, and $PZ_{78}$ in [16] to the trivial diagram; in contrast, these require adding at least three crossings if one uses only Reidemeister moves.

## A Lower Bound on Computational Complexity

In this section, we show that the worst-case complexity of Find Diagram is at least exponential by exhibiting inputs where any output must be exponentially large. Specifically, let $F_n$ be the Fibonacci sequence and $\alpha =\bigl (1+\sqrt{5}\bigr )/2\approx 1.618$ be the golden ratio. Let $K_n$ be the torus knot $T(F_n,F_{n-1})$. Then:

### Theorem 9.1

There is an ideal triangulation of $E(K_n)$ with *O*(*n*) tetrahedra, but the minimum crossing number of $K_n$ is $F_{n-1}(F_n-1)\sim \alpha ^{2n-1}/5$.

We now give the short proof, which is similar to the analysis of the complexity of a normal meridional disk in a minimal triangulation of a layered solid torus in [38, Sect. 5].

### Proof

Set $p=F_n$ and $q=F_{n-1}$. The claim about the crossing number of $K_n$ is [49, Prop. 7.5]. For the ideal triangulation of $E(K_n)$, first we construct a 1-vertex triangulation ${\mathcal T}$ of $S^3$ where $K_n$ is an edge. We do this by gluing two layered solid tori (see Sect. 3) to form the genus-1 Heegaard splitting of $S^3$ so that the torus knot $K_n$ is an edge on their common interface. Since the partial quotients of the continued fraction expansion of *p*/*q* are all 1, this requires only *O*(*n*) tetrahedra, see [40]. Now, take the second barycentric subdivision of ${\mathcal T}$ and delete the interior of the link of $K_n$ to get a triangulation ${\mathcal T}'$ of the compact manifold $E(K_n)$ with *O*(*n*) tetrahedra. Finally, crush as in [37, Thm. 7.1] to get an ideal triangulation $\mathring{{\mathcal T}}$ for $E(K_n)$ with no more tetrahedra than ${\mathcal T}'$. $\square$

## Implementation and Initial Experiments

We implemented our algorithm in Python, building on the pure-Python t3mlite library for $3$-manifold triangulations that is part of SnapPy [18]. We also used SnapPy’s C kernel to produce the layered filled triangulation ${\mathcal T}$ of Sect. 2.3 from the input ideal triangulation $\mathring{{\mathcal T}}$. The needed linear algebra over ${\mathbb Q}$ was handled by PARI.^1^ Not including these libraries, our implementation consists of 1800 lines of Python code. We had to put considerable effort into optimization to handle examples as large as that shown in Fig. 3. Our code and data is archived at [20] and has been incorporated into version 3.1 of SnapPy [18].

To validate our implementation, we applied it to two sample sets, one where the inputs were small and one where the best-possible outputs were small. The first, $\mathcal C{\mathcal K}$, is the 1267 hyperbolic knots whose exteriors have ideal triangulations with at most nine tetrahedra [5, 19]. The second, ${\mathcal S}{\mathcal K}$, consists of 1000 knots with minimal crossing numbers between 10 and 19. Specifically, ${\mathcal S}{\mathcal K}$ has 100 knots for each crossing number in that range, which were selected at random from all the hyperbolic nonalternating knots with that crossing number [12]; the exception is that there are only 41 such 10-crossing knots, so 59 alternating 10-crossing knots were used as well. (Alternating knots have unusually close connections between their diagrams and exteriors, so were excluded as possibly being an easy case for Find Diagram.)

**Fig. 24:**
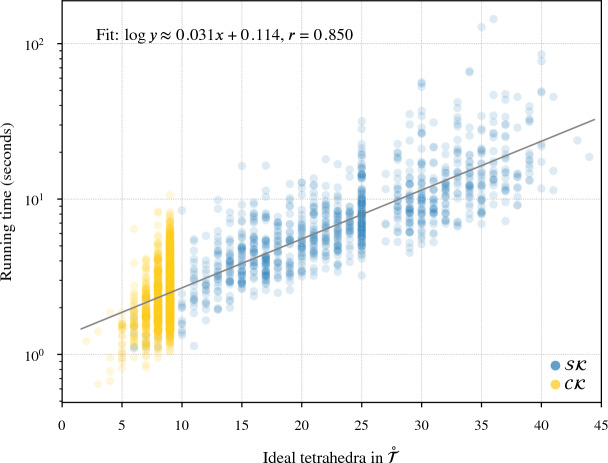
Mean running time for the 2267 knot exteriors in ${\mathcal S}{\mathcal K}$ and $\mathcal C{\mathcal K}$ appears exponential with small base, roughly $O(1.07^n)$. Compare Fig. 29 on the growth of the number of arcs in ${\mathcal T}_0$

**Fig. 25:**
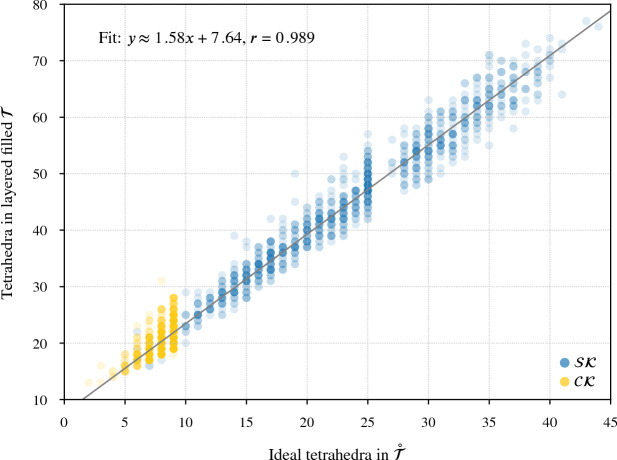
The number of tetrahedra in the layered filled ${\mathcal T}$ compared to the input ideal $\mathring{{\mathcal T}}$

**Fig. 26:**
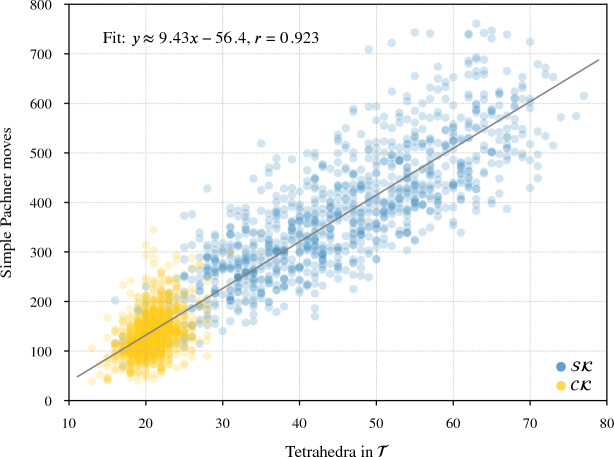
The number of *simple* Pachner moves used to transform the layered filled triangulation ${\mathcal T}$ into the base triangulation ${\mathcal T}_0$ is generically linear in the size of ${\mathcal T}$

**Fig. 27:**
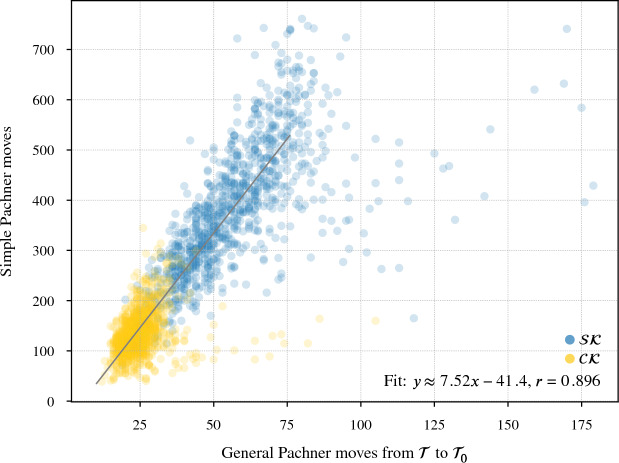
This plot shows the increase in the number of Pachner moves when we factor the $2\,{\rightarrow }\,0$ moves into simple Pachner moves. The regression line is based on points with $x<75$

**Fig. 28:**
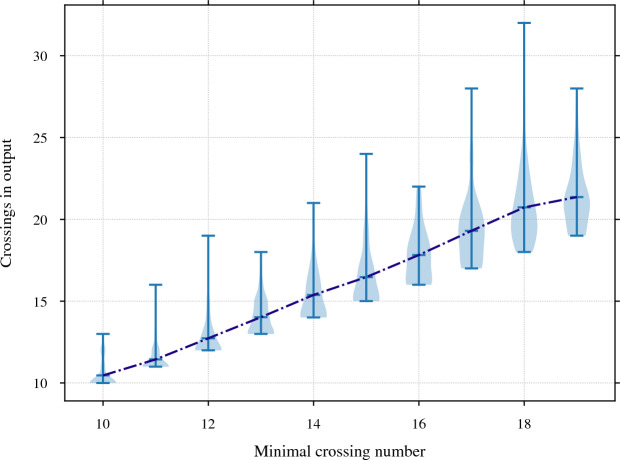
For the knots in ${\mathcal S}{\mathcal K}$, grouped by minimum crossing number, the number of crossings in the diagram output by our program. The dotted line indicates the mean

Our program found diagrams for all 2267 of these exteriors. The running time was under 20 seconds for $96.7\,\%$ of them, with a max of 2.5 min (CPUs were Intel Xeon E5-2690 v3 at 2.6 GHz with 4GB of memory per core, ca. 2014); see Fig. 24. The input ideal triangulations $\mathring{{\mathcal T}}$ had between 2 and 44 tetrahedra, and the resulting layered filling triangulation ${\mathcal T}$ had between 13 and 77 tetrahedra (mean of 31.5), typically $60\,\%$ larger than $\mathring{{\mathcal T}}$; see Fig. 25. The sequence of simple Pachner moves used to reduce ${\mathcal T}$ to ${\mathcal T}_0$ had length between 39 and 761 (mean of 241.0), see Fig. 26; this was typically 7.5 times longer than the initial sequence of Pachner moves that included $2\,{\rightarrow }\,0$ moves (Fig. 27). For the knots in ${\mathcal S}{\mathcal K}$, we compare the size of the output diagram to the minimal crossing number in Fig. 28; the output matched the crossing number for $42.1\,\%$ of these exteriors, and it was within 3 for $87.8\,\%$. For $\mathcal C{\mathcal K}$, the maximal number of crossings in the output was 303, with mean output crossing number 65.9, and median output crossing number 40.

**Fig. 29:**
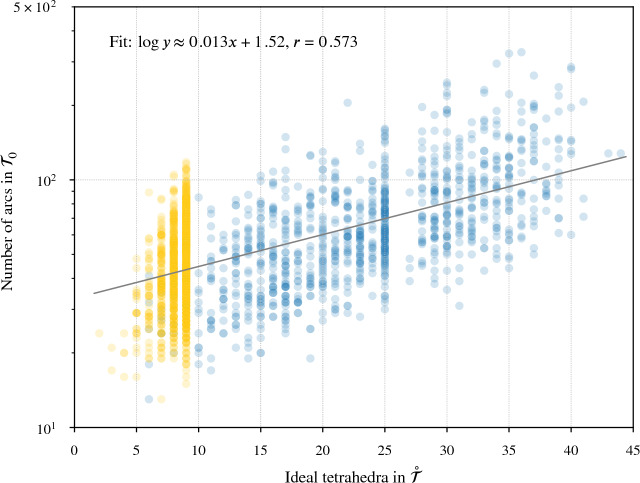
The number of barycentric arcs when we arrive at ${\mathcal T}_0$ appears exponential in the size of the input $\mathring{{\mathcal T}}$, roughly $O(1.03^n)$

## Applications

### Congruence Links

**Fig. 30:**
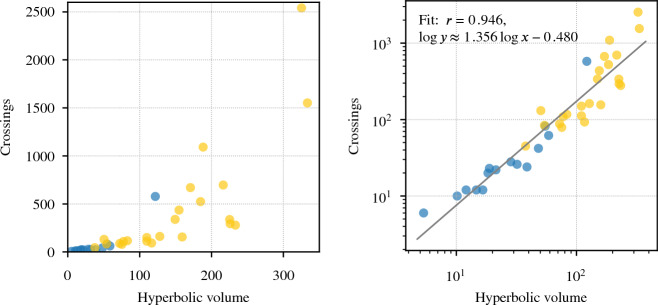
The 38 known link diagrams whose exteriors are principal congruence arithmetic; blue are the 15 from [7], yellow are new. The plots are the same except for the scales on the axes. The regression at right predicts that our algorithm would produce a diagram for the link for the largest such exterior with about 9000 crossings

Powerful tools from number theory apply to the special class of arithmetic hyperbolic $3$-manifolds. Thurston asked which link exteriors are in the subclass of principal congruence arithmetic manifolds; this was resolved in [6]: there are exactly 48 such exteriors. These 48 have hyperbolic volumes in [5.33348, 1365.37] and ideal triangulations with between 6 and 1526 tetrahedra. Link diagrams for 15 of these 48 had previously been found by ad hoc methods [7]. Our program has found diagrams for 23 more, including Figs. 2 and 3; collectively, we now have links for the 38 such exteriors of smallest volume, see Fig. 30.

**Fig. 31:**
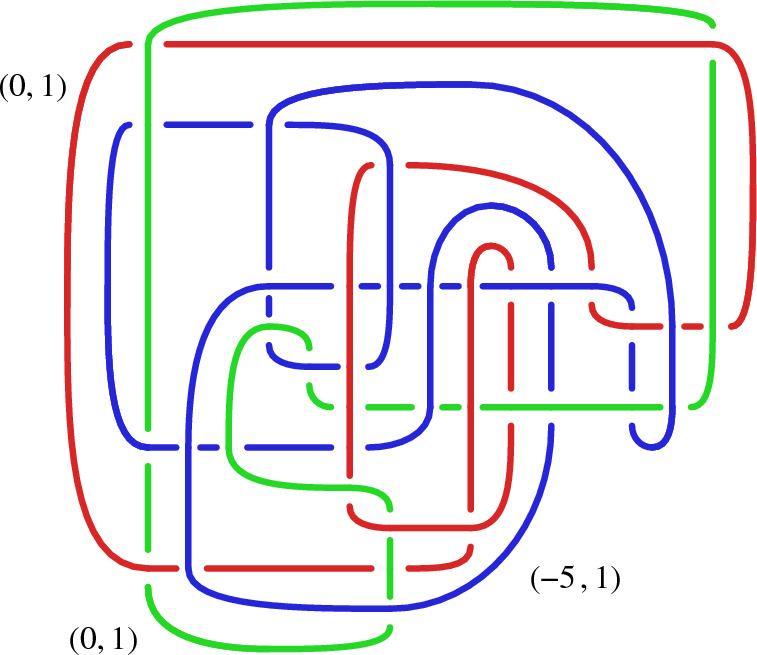
A Dehn surgery description of the Seifert–Weber dodecahedral space

### Dehn Surgery Descriptions

Every closed orientable 3-manifold is a Dehn filling on some link exterior in $S^3$ [58, Chap. 9], and such *Dehn surgery descriptions* play a key role in both theory and practice. However, finding a Dehn surgery description from e.g. a triangulation can be extremely challenging. Thurston observed experimentally that, starting with a closed hyperbolic $3$-manifold, one frequently arrives at a link exterior by repeatedly drilling out short closed geodesics, see page 516 of [2]. Combining this with our algorithm for Find Diagram gives an effective tool for finding Dehn surgery descriptions given a triangulation. We applied this to the Seifert–Weber dodecahedral space, which is an old example [68] still of much current interest [9, 14, 43]. The resulting description in Fig. 31 seems to be the first such published; a different description appeared subsequently in [4]. One could likely use a similar technique to find Dehn surgery descriptions of a nonhyperbolic 3-manifold, for example by first removing a complicated knot whose complement is hyperbolic [50] and then proceeding as before, or by using some other method to select promising curves to drill out.

### Knots with the Same 0-Surgery

The 0-surgery $Z(K)$ on a knot *K* is the unique Dehn filling *N* of $E(K)$ where $H_1(N;{\mathbb Q})\ne 0$. Pairs of knots *K* and $K'$ with $Z(K)$ homeomorphic to $Z(K')$ are of much interest in low-dimensional topology. Most strikingly, if such a pair *K* and $K'$ exist with *K* slice (i.e., bounds a smooth $D^2$ in $D^4$) and the Rasmussen *s*-invariant of $K'$ is nonzero, then the smooth 4-dimensional Poincaré conjecture is false. That is, there would exist a 4-manifold that is homeomorphic but not diffeomorphic to $S^4$. See [27, 45] for a general discussion, and also [56] for an important recent result using pairs with $Z(K)\cong Z(K')$. There are many techniques for constructing families of such pairs, which have been unified by the red-blue-green link framework of [45]. However, given a particular *K*, a practical algorithm to search for $K'$ with the same 0-surgery has been lacking. When *Z*(*K*) is hyperbolic, we attack this as follows. First, find the short closed geodesics in *Z*(*K*) using [32]. Then drill out each geodesic in turn, and test if the resulting manifold $\mathring{M}'$ has a Dehn filling which is $S^3$; if it does, use our algorithm for Find Diagram to $\mathring{M}'$ to get a diagram for $K'$.

**Fig. 32:**
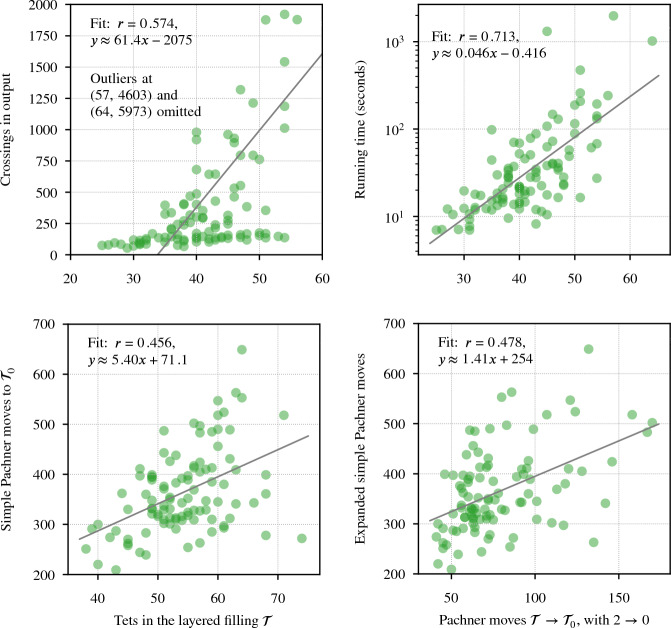
Data on the 100 knot exteriors from Sect. 11.3

Figure 32 shows the result of applying our algorithm to 100 pairs $(K,\gamma )$ where *K* is a knot with at most 18 crossings and $\gamma$ is a short closed geodesic in $Z(K)$ whose exterior is also that of a knot $K'$ in $S^3$. In all cases, we were able to recover a diagram for $K'$, and these were more challenging on average than the examples in Sect. 10.

## Conclusion and Open Questions

To assess the practical effectiveness of our algorithm for Find Diagram, it is worth considering how large a link diagram can be used as input for a subsequent computation. Many key link invariants, such as the Alexander polynomial and the Seifert genus, can be computed directly (and at least as efficiently) from a triangulation of the link exterior as from the diagram, so we focus on those that require a diagram to compute. The Jones polynomial is among the simpler such “truly diagrammatic” invariants, and it is #P-hard [41] to compute, though it is fixed-parameter tractable in the tree-width of the diagram [44]. Even the very best implementations for computing the Jones polynomial are unable to handle most diagrams with more than 200 crossings [62]. More refined invariants, such as knot Floer homology [53, 66] and Khovanov homology [8, 61, 62], are typically impractical above 50–100 crossings, or even less depending on the precise variant.

As Fig. 32 demonstrates, our implementation easily finds link diagrams which are too big to allow computation of such diagrammatic invariants. While these diagrams presumably do not minimize the number of crossings, we expect they are close enough that the point still stands. Alternatively, recall the diagram in Fig. 2 produced by our algorithm has 294 crossings, and, by volume considerations, any diagram of this link must have at least 66 crossings, and that alone would push the limits of many diagrammatic calculations.

Given the practical effectiveness of our implementation of Find Diagram, we have incorporated it as a standard feature of SnapPy [18], so that it can be widely used. We conclude with several open questions.

1. To what extent can the mean running time of $O(1.07^n)$ be reduced? While Theorem 9.1 shows that the worst case running time must be at least exponential, it is not implausible that the mean running time is polynomial in the size of the *output*. The key issue is that the number of arcs in the base triangulation ${\mathcal T}_0$ is currently exponential in the size of both the input *and* the output, compare Fig. 29. In our current implementation, as the size of the input exceeds 40 tetrahedra, the computation time becomes dominated by the final diagram simplification step. This is despite the very favorable $O(n^{1.5})$ performance of our diagram simplification algorithm (Fig. 23). This again suggests we need to simplify the barycentric link more during the Pachner move steps.
2. To reduce the number of arcs when applying Pachner moves, one could consider additional local PL simplification moves, or try the current moves in larger balls in ${\mathcal T}$ made up of several tetrahedra. We tried using the straighten simplification method inside the octahedron formed during the $4\,{\rightarrow }\,4$ move, but, in the examples we tested, this had the surprising effect of increasing the number of arcs at the final stage of the algorithm. We also tried adding more complicated PL simplifications moves beyond straighten and push, but these had minimal effect.
3. It would be interesting to see if knot energy minimization, see [52] and [64], provides a practical way to simplify configurations of arcs within tetrahedra or to simplify the final link in ${\mathbb R}^3$ before projecting.
4. Our algorithm provides a new way to move in the space of diagrams for a given link: take a link diagram, produce a triangulation of the exterior, apply Pachner moves to modify the triangulation, then apply our algorithm to this new triangulation to obtain a new diagram of the link. It would be interesting to see if this approach effectively finds simple diagrams of links, or allows us to produce distinct simple diagrams that require many Reidemeister moves (or many additional crossings) to go between.
5. Is it possible to find diagrams for the remaining ten congruence link exteriors discussed in Sect. 11.1?

## Supplementary Information

Our code and data is archived at [20]. The earlier proceedings version of this paper is [21].

## Footnotes

1 PARI/GP v. 2.11.4 (2020). The PARI Group, University of Bordeaux. http://pari.math.u-bordeaux.fr.

## References

[1] Adams CC (1994). The Knot Book: An Elementary Introduction to the Mathematical Theory of Knots.

[2] Adams CC (1999). Isometric cusps in hyperbolic $$3$$-manifolds. Mich. Math. J..

[3] Adams, C.: Triple crossing number of knots and links. J. Knot Theory Ramif. **22**(2), # 1350006 (2013)

[4] Baker, K.L.: A sketchy surgery description of the Seifert–Weber Dodecahedral space (2021). https://sketchesoftopology.wordpress.com/2021/12/09/a-sketchy-surgery

[5] Baker, K.L., Kegel, M.: Census L-space knots are braid positive, except one that is not. Algebr. Geom. Topol. https://msp.org/soon/coming.php?jpath=agt

[6] Baker MD, Goerner M, Reid AW (2019). All principal congruence link groups. J. Algebra.

[7] Baker, M.D., Goerner, M., Reid, A.W.: All known principal congruence links (2019). arXiv:1902.04426

[8] Bar-Natan D (2007). Fast Khovanov homology computations. J. Knot Theory Ramif..

[9] Bering, E.A. IV: Surgery diagram for the Seifert–Weber space. MathOverflow, question # 137101 (2013). https://mathoverflow.net/q/137101

[10] Burton, B.A.: The Pachner graph and the simplification of $$3$$-sphere triangulations. In: 27th Annual Symposium on Computational Geometry (Paris 2011), pp. 153–162. ACM, New York (2011)

[11] Burton, B.A.: Computational topology with Regina: algorithms, heuristics and implementations. In: Geometry and Topology Down Under. Contemporary Mathematics, vol. 597, pp. 195–224. American Mathematical Society, Providence (2013)

[12] Burton, B.A.: The next 350 million knots. In: 36th International Symposium on Computational Geometry (2020). Leibniz Int. Proc. Inform., vol. 164, # 25. Leibniz-Zent. Inform., Wadern (2020)

[13] Burton BA (2023). The cusped hyperbolic census is complete. Trans. Am. Math. Soc..

[14] Burton BA, Rubinstein JH, Tillmann S (2012). The Weber–Seifert dodecahedral space is non-Haken. Trans. Am. Math. Soc..

[15] Burton, B.A., Budney, R., Pettersson, W.: Regina: software for low-dimensional topology. http://regina-normal.github.io/

[16] Burton, B.A., Chang, H.-Ch., Löffler, M., Maria, C., de Mesmay, A., Schleimer, S., Sedgwick, E., Spreer, J.: Hard diagrams of the unknot. Exp. Math. (2023). 10.1080/10586458.2022.2161676

[17] Champanerkar, A., Kofman, I., Mullen, T.: The 500 simplest hyperbolic knots. J. Knot Theory Ramif. **23**(12), # 1450055 (2014)

[18] Culler, M., Dunfield, N.M., Goerner, M., Weeks, J.R.: SnapPy, a computer program for studying the geometry and topology of $$3$$-manifolds, v. 3.1 (2022). https://snappy.computop.org

[19] Dunfield, N.M.: A census of exceptional Dehn fillings. In: Characters in Low-Dimensional Topology (Montréal 2018). Contemporary Mathematics, vol. 760, pp. 143–155. American Mathematical Society, Providence (2020)

[20] Dunfield NM, Obeidin M, Rudd CG (2022). Code and data for computing a link diagram from its exterior. Harvard Dataverse.

[21] Dunfield, N.M., Obeidin, M., Rudd, C.G.: Computing a link diagram from its exterior. In: 38th International Symposium on Computational Geometry (Berlin 2022). Leibniz Int. Proc. Inform., vol. 224, # 37. Leibniz-Zent. Inform., Wadern (2022)

[22] Dynnikov IA (1999). Three-page approach to knot theory. Encoding and local moves. Funct. Anal. Appl..

[23] Dynnikov IA (2006). Arc-presentations of links: monotonic simplification. Fund. Math..

[24] Dynnikov, I., Sokolova, V.: Multiflypes of rectangular diagrams of links. J. Knot Theory Ramif. **30**(6), # 2150038 (2021)

[25] Flapan E (2000). When Topology Meets Chemistry. Outlooks.

[26] Flapan E, He A, Wong H (2019). Topological descriptions of protein folding. Proc. Natl. Acad. Sci. U.S.A..

[27] Freedman M, Gompf R, Morrison S, Walker K (2010). Man and machine thinking about the smooth $$4$$-dimensional Poincaré conjecture. Quant. Topol..

[28] Gordon, C.McA., Luecke, J.: Knots are determined by their complements. J. AMS **2**(2), 371–415 (1989)

[29] Greene JE (2017). Alternating links and definite surfaces. Duke Math. J..

[30] Haken W (1961). Theorie der Normalflächen. Acta Math..

[31] Hass, J., Lagarias, J.C., Pippenger, N.: The computational complexity of knot and link problems. J. ACM **46**(2), 185–211 (1999)

[32] Hodgson CD, Weeks JR (1994). Symmetries, isometries and length spectra of closed hyperbolic three-manifolds. Exp. Math..

[33] Hoste J, Thistlethwaite M, Weeks J (1998). The first $$1{,}701{,}936$$ knots. Math. Intell..

[34] Howie JA (2017). A characterisation of alternating knot exteriors. Geom. Topol..

[35] Huszár, K., Spreer, J.: $$3$$-manifold triangulations with small treewidth. In: 35th International Symposium on Computational Geometry (Portland 2019). Leibniz Int. Proc. Inform., vol. 129, # 44. Leibniz-Zent. Inform., Wadern (2019)

[36] Ivanov SV (2008). The computational complexity of basic decision problems in $$3$$-dimensional topology. Geom. Dedicata.

[37] Jaco W, Rubinstein JH (2003). $$0$$-efficient triangulations of $$3$$-manifolds. J. Differ. Geom..

[38] Jaco, W., Rubinstein, J.H.: Layered-triangulations of $$3$$-manifolds (2006). arXiv:math/0603601

[39] Jaco W, Rubinstein JH (2014). Inflations of ideal triangulations. Adv. Math..

[40] Jaco W, Sedgwick E (2003). Decision problems in the space of Dehn fillings. Topology.

[41] Jaeger F, Vertigan DL, Welsh DJA (1990). On the computational complexity of the Jones and Tutte polynomials. Math. Proc. Camb. Philos. Soc..

[42] Kuperberg G (2019). Algorithmic homeomorphism of $$3$$-manifolds as a corollary of geometrization. Pac. J. Math..

[43] Lin F, Lipnowski M (2022). Monopole Floer homology, eigenform multiplicities, and the Seifert–Weber dodecahedral space. Int. Math. Res. Not..

[44] Makowsky JA (2005). Coloured Tutte polynomials and Kauffman brackets for graphs of bounded tree width. Discrete Appl. Math..

[45] Manolescu, C., Piccirillo, L.: From zero surgeries to candidates for exotic definite four-manifolds (2021). arXiv:2102.04391

[46] Matveev, S.: Algorithmic Topology and Classification of $$3$$-Manifolds. Algorithms and Computation in Mathematics, vol. 9. Springer, Berlin (2007)

[47] de Mesmay, A., Rieck, Y., Sedgwick, E., Tancer, M.: The unbearable hardness of unknotting. Adv. Math. **381**, # 107648 (2021)

[48] Mijatović A (2003). Simplifying triangulations of $$S^3$$. Pac. J. Math..

[49] Murasugi K (1991). On the braid index of alternating links. Trans. Am. Math. Soc..

[50] Myers R (1982). Simple knots in compact, orientable $$3$$-manifolds. Trans. Am. Math. Soc..

[51] Obeidin, M.: Link simplification code for Spherogram. https://github.com/3-manifolds/Spherogram/blob/master/spherogram_src/links/simplify.py

[52] O’Hara J (1991). Energy of a knot. Topology.

[53] Ozsváth P, Szabó Z (2019). Bordered knot algebras with matchings. Quant. Topol..

[54] Pachner U (1991). P.L. homeomorphic manifolds are equivalent by elementary shellings. Eur. J. Combin..

[55] Peddada, S.R.T., Dunfield, N.M., Zeidner, L.E., James, K.A., Allison, J.T.: Systematic enumeration and identification of unique spatial topologies of 3D systems using spatial graph representations. In: 47th Design Automation Conference (2021). International Design Engineering Technical Conferences & Computers and Information in Engineering Conference, vol. 3A, # V03AT03A042. ASME, New York (2021)

[56] Piccirillo L (2020). The Conway knot is not slice. Ann. Math..

[57] Piergallini, R.: Standard moves for standard polyhedra and spines. In: 3rd National Conference on Topology (Trieste 1986). Rend. Circ. Mat. Palermo Suppl., vol. 18, pp. 391–414. Circolo Matematico di Palermo, Palermo (1988)

[58] Rolfsen, D.: Knots and Links. Mathematics Lecture Series, vol. 7. Publish or Perish, Houston (1990)

[59] Schirra, S.: Robustness and precision issues in geometric computation. In: Handbook of Computational Geometry, pp. 597–632. North-Holland, Amsterdam (2000)

[60] Schleimer, S.: Sphere recognition lies in NP. In: Low-Dimensional and Symplectic Topology (Athens 2009). Proc. Sympos. Pure Math., vol. 82, pp. 183–213. American Mathematical Society, Providence (2011)

[61] Schütz D (2021). A fast algorithm for calculating $$S$$-invariants. Glasg. Math. J..

[62] Schütz, D.: Knotjob (2022, blue version). https://www.maths.dur.ac.uk/users/dirk.schuetz/knotjob.html

[63] Segerman H (2017). Connectivity of triangulations without degree one edges under $$2$$-$$3$$ and $$3$$-$$2$$ moves. Proc. Am. Math. Soc..

[64] Simon JK (1994). Energy functions for polygonal knots. J. Knot Theory Ramif..

[65] Sundberg C, Thistlethwaite M (1998). The rate of growth of the number of prime alternating links and tangles. Pac. J. Math..

[66] Szabó, Z.: Knot Floer homology calculator (2022). https://web.math.princeton.edu/~szabo/HFKcalc.html

[67] Tillmann S (2008). Normal surfaces in topologically finite 3-manifolds. Enseign. Math..

[68] Weber C, Seifert H (1933). Die beiden Dodekaederräume. Math. Z..

[69] Weeks JR (1993). Convex hulls and isometries of cusped hyperbolic $$3$$-manifolds. Topol. Appl..

[70] Weeks, J.R.: Source code file close_cusps.c for SnapPea, v. 2.5 (circa 1995). https://github.com/3-manifolds/SnapPy/blob/master/kernel/kernel_code/

[71] Weeks, J.: Computation of hyperbolic structures in knot theory. In: Handbook of Knot Theory, pp. 461–480. Elsevier, Amsterdam (2005)

[72] Zentner R (2018). Integer homology $$3$$-spheres admit irreducible representations in SL$$(2,{\mathbb{C} })$$. Duke Math. J..

